# 3D geological and petrophysical modeling of Alam El-Bueib Formation using well logs and seismic data in Matruh Field, northwestern Egypt

**DOI:** 10.1038/s41598-024-56825-5

**Published:** 2024-03-21

**Authors:** Walaa A. Ali, Amr S. Deaf, Taher Mostafa

**Affiliations:** 1 Petroleum Geology Department, Faculty of Petroleum and Mining Sciences, Matrouh University, Matrouh, Egypt; 2 Geology Department, Faculty of Science, Assiut University, Assiut, 71516 Egypt; 3 Geology Department, Faculty of Science, Al-Azhar University, Nasr City, 11884 Cairo Egypt

**Keywords:** 3D static reservoir modeling, Alam El-Bueib, Reservoir evaluation,, Matruh oil field, Egypt, Energy science and technology, Engineering, Solid Earth sciences, Geochemistry, Geology, Geophysics, Sedimentology

## Abstract

There are several productive petroleum fields in the North Western Desert (WD) of Egypt, which received extensive investigations regarding their petroleum potential. However, a few studies tackled the Matruh Oil Field, which contains the oil prolific Early Cretaceous Alam El-Bueib Formation (AEB Fm) reservoir. The reservoir intervals of the AEB Fm show substantial lithological variations across the basin. Therefore, it is necessary to analyze the vertical and lateral distributions in terms of their lithological and petrophysical properties. To achieve this objective, wireline logs of four wells and 20-2D seismic lines were used to construct a depth-structure contour map for the studied part of the field. This map was used to build the field’s structure model and to identify the fault patterns in the basin through several seismic lines. Analyses of well logs data and lithology were used to estimate the petrophysical properties of AEB sandstone units AEB-1, AEB-3A, AEB-3C, and AEB-6. Results show that the AEB-6 Unit is the most promising hydrocarbon-bearing unit. It has a net pay of 20–160 feet, a shale volume of 5–20%, an effective porosity of 14–20%, and a hydrocarbon saturation of 70–88%. The structure-depth maps indicate a number of normal faults with two principal NE-SW and NW–SE trends, which probably act as structural traps in the Matruh Oil Field. The constructed structure-depth maps and calculated petrophysical parameters were used to build a three-dimensional reservoir model. A blind well was used to validate the accuracy and reliability of the facies, porosity, and saturation models for the AEB Fm units, ensuring a good match between log-derived data and built models. The AEB Fm shows regional heterogeneous variations in its petrophysical characteristics. It exhibits unconventional reservoir characteristics in a N–S direction and conventional reservoir characteristics in an E–W direction. This observed heterogeneity shows the need to carry out further investigations to comprehensively assess the hydrocarbon potential of AEB Fm in different areas of the Matruh Basin.

## Introduction

**Figure 1:**
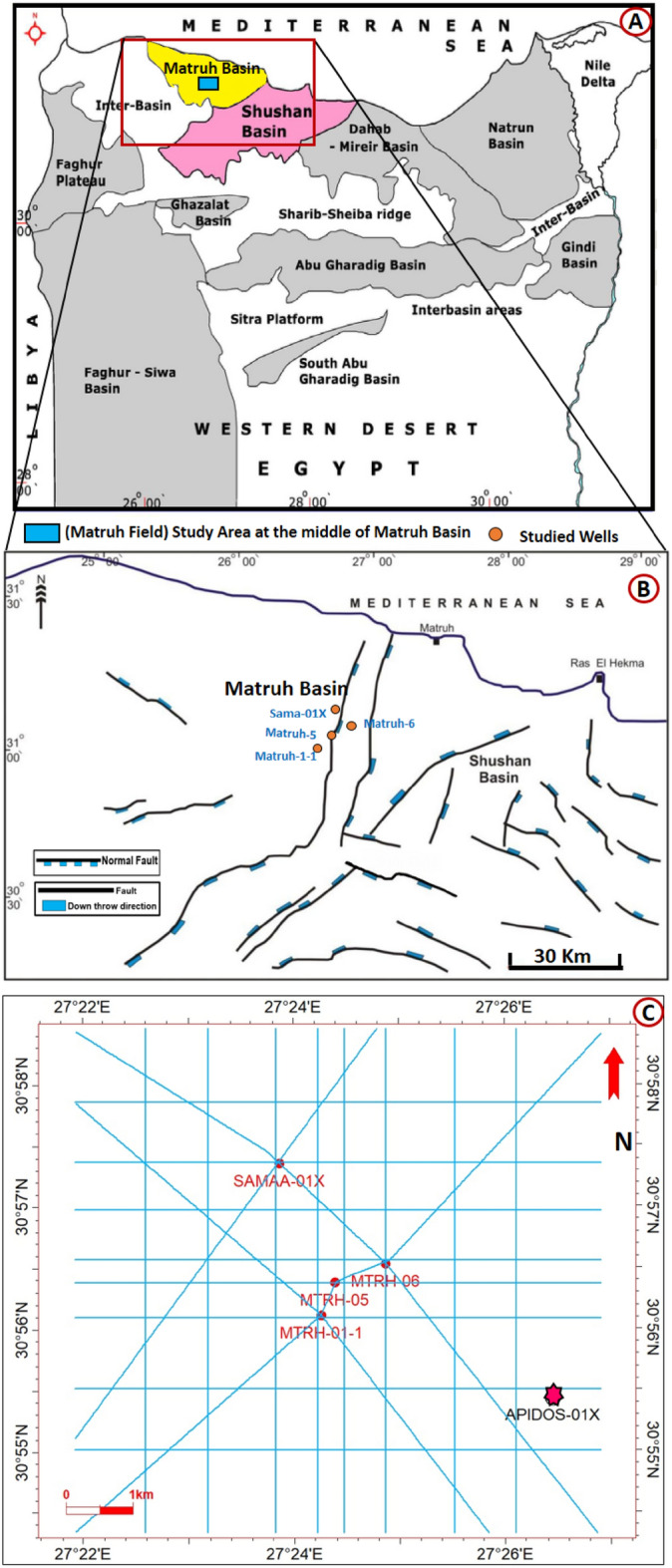
(**A**) Location map of the Matruh-Shushan Basin in the Western Desert, showing the main Mesozoic basins and study area of Matruh Field (blue rectangular) (**B**) The enlarged area shows the pattern of structures and main faults in the Matruh and Shushan Basins, North Western Desert, Egypt, and the location of the studied wells (**C**) Location map of the studied wells in Matruh Field within seismic lines and the location of the validation well (Apidose-1X) for modeling. (This figure is modified after^7^, Copyright © 2013 John Wiley & Sons, Ltd).

Since global oil demand is rising, it is expected to surpass pre-pandemic levels, reaching 101.6 million barrels per day (BPD) in 2023^1^. Boosting the hydrocarbon supply to meet that demand is essential. To increase the supply of oil, exploration and production processes should be conducted with caution. More attention should be paid to oil field exploration having potential reservoirs. One of the significant contributors to Egypt’s oil production is the northern region of the Western Desert. The Matruh Basin exploration area has 23 billion barrels of oil equivalent (BBOE) with a low risk of hydrocarbon accumulation^2^**.** The Matruh Basin is situated in the northern region of the Western Desert (Fig. 1) and has a substantial hydrocarbon system of Cretaceous origin, making it one of the most significant petroliferous areas in northern Egypt^3,4^. The Matruh Oil Field, the focus of this study is situated within the middle Matruh Basin at latitudes 30° 55′ 41″ and 30° 57′ 37″ N, and longitudes 27° 23′ 18″, 27° 25′ 18″ E (Fig. 1)^5^. The Matruh Field **(**Fig. 1A**)** is approximately 28.47 km^2^ and is located 45 km southeast of Matruh Town. The Matruh Field was discovered in 1991, when the Matruh 1-1 well was drilled and the logs showed hydrocarbon-bearing sandstone reservoirs of the Middle Jurassic Khatatba Formation. The maximum oil production from the Western Desert is derived from its northern basins, particularly the Matruh Basin, of which 70% comes from the Jurassic and Cretaceous sandstone reservoirs^6^.

Although several publications investigated the hydrocarbon potential of the Middle Jurassic Khatatba and Lower Cretaceous Alam El-Bueib formations in the northern part of WD, including^2,7–20^, just a few studies^15,21–24^ focused on the Matruh Basin. Only three published studies dealt with the structural modelling of potential traps and/or reservoir quality of the Alam El-Bueib Formation in the Matruh Basin. The first study focused on the fault systems in the Jade Oil Field in western Matruh Basin^25^ and suggested that the main structural trap is an anticlinal fold that was moving in a NE-SW direction along a reversed fault. They suggested that this fault was developed during the Jurassic period and was rejuvenated and reversed during the Late Cretaceous regional basin inversion.

This was probably related to the Tethyan rifting and NE drifting of the African plate towards the Eurasian plate^19^, which caused major uplifting and basin inversion of multiple regions in the northwestern area of Egypt, such as the Matruh Basin. The second study focused on assessing the reservoir and source potential of the Alam El-Bueib Formation through a single well^26^. The authors reached a conclusion that the Alam El-Bueib Formation exhibits important reservoir characteristics and lower source rock potential. They identified four main reservoir intervals made up of sandstones and carbonates, where clastic units show significant variations in the shale volume (6.5–56.1%). These reservoirs possess fair to good porosity characters (4.0–7.1%), varying water saturation (11.3–84.4%) and low to good hydrocarbons saturation (11.3–88.7%) values. The third study focused on the reservoir quality of the Alam El-Bueib Formation through four wells in the Jade Oil Field^22^. This study suggested that the sandstones of the AEB-3G and AEB-6 members are good reservoirs. They show good porosity (12.5–16%), high hydrocarbon saturation (up to 80%), and fair movable hydrocarbon volumes (up to 15%), but with varying water saturation values (4–22%) and shale volumes (8–14%). This study suggested that the AEB-3G and AEB-6 members of the Alam El-Bueib Formation have actual hydrocarbon accumulations. Furthermore, the intra- and inter-basinal stratigraphic correlations along the Matruh Basin and its adjacent Shushan Basin indicate that the Alam El-Bueib Formation shows evident lateral and vertical facies changes^27^. This indicates that the reservoir quality of the Alam El-Bueib Formation is affected by volume and vertical/lateral distributions of shale deposits almost along the entire formation on local and regional scales in the Matruh Basin.

Overall, the above-mentioned studies show that different parts of the Matruh Basin exhibit different vertical/lateral facies and petrophysical characteristics and possess various structural traps of different styles. This indicates that the reservoir potential of the Lower Cretaceous Alam El-Bueib Formation is still underexplored in the Matruh Basin. Therefore, the current work was designed to shed more light on the reservoir potential of the Alam El-Bueib Formation in another petroleum productive area of the Matruh Basin. To the best of our knowledge, this research study is the initial examination conducted on a regional level, in which many wells were utilized to assess the reservoir quality of the Alam El-Bueib Formation in the Matruh Oil Field. This was achieved by constructing a depth-structure contour map for the Matruh Field using 2D seismic data and wireline logs of four wells. In addition, the identification of the fault patterns penetrating the basin was made using seismic data interpretation. Analysis of well logs data was also made to recognize the lateral and vertical variations in the lithological and petrophysical parameters of the AEB Formation’ units (AEB-1, AEB-3A, AEB-3C, and AEB-6). This was supplemented with an analysis of the Rock–Eval data to assess the reservoir and source rock potential of shaly sandstone facies within the AEB Formation.

## The geological settings

**Figure 2:**
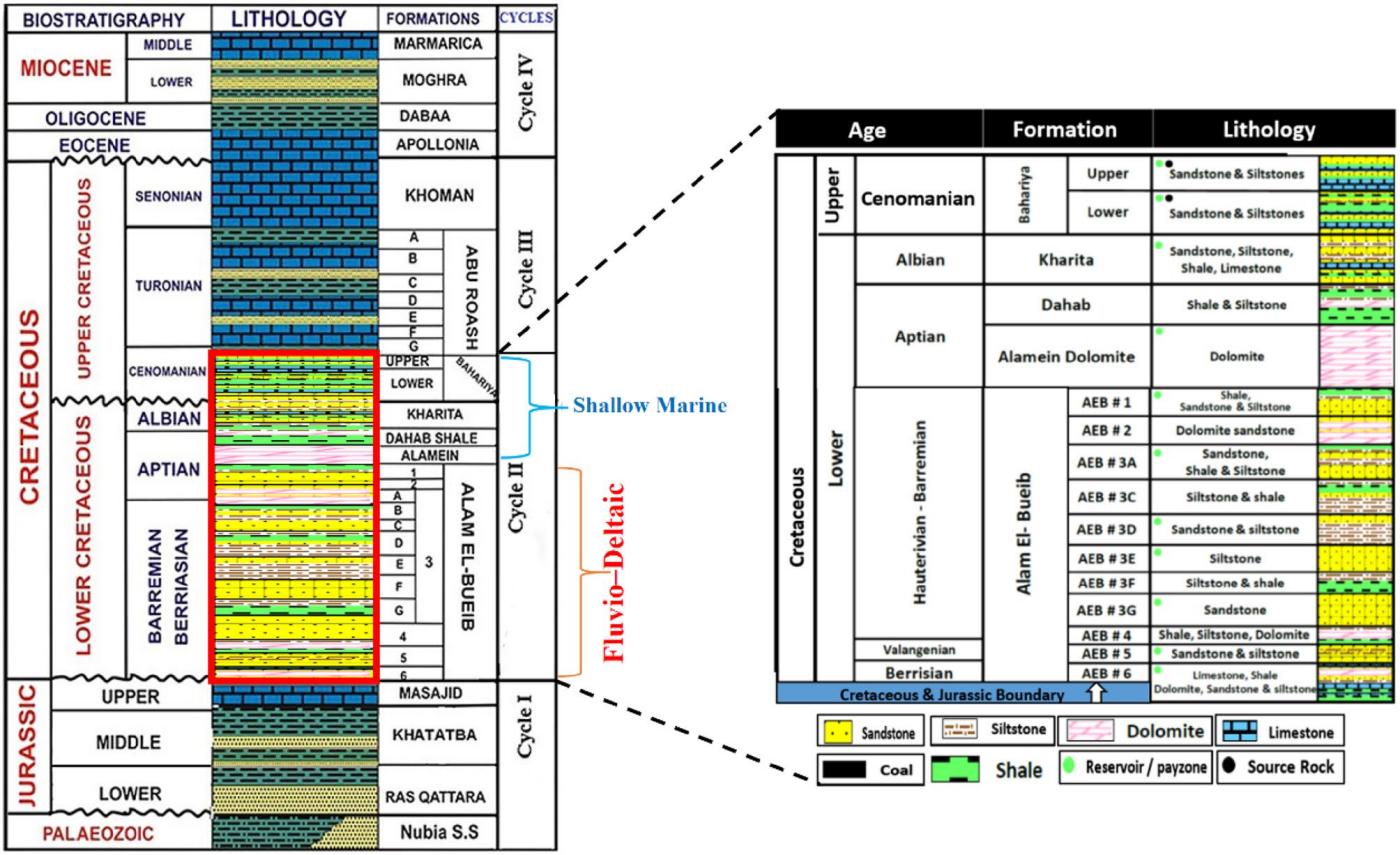
Stratigraphic column showing the formations and their lithology encountered in the Western Desert of Egypt (Modified from^33^).

The Matruh Basin lies between longitudes 26° and 27° 30' E and 31° and latitudes 31° 17' N^3,18–20^ and represents a part of the unstable tectonic realm, which encompasses the entire northern part of Egypt including the North Western Desert of Egypt. The Mesozoic tectonic activities in the North Western Desert region resulted in the formation of several basins, where depositional history of the Mesozoic sedimentary successions were mainly affected by these tectonics. The trapped hydrocarbon accumulations are largely connected to the Jurassic and Cretaceous tectonostratigraphic evolution of the Matruh Basin. This evolution resulted in the formation of several reservoir and seal systems. The tectonic evolution of northern Egypt indicates three main tectonic events^10,12^ (Fig. 2).

The first event resulted in the formation of the Paleozoic-Early Mesozoic (Triassic) fault with a NW to WNW trend. The second event took place during the Late Mesozoic (Late Cretaceous) and resulted in the formation of a ENE-trending series of faulted anticlines commonly known as the “Syrian Arc System”^19,28^. The third event occurred during the Early Paleogene (Late Eocene and Early Oligocene) and formed the NW-trending Gulf of Suez and NNE-trending Aqaba faults. Several investigations of the tectonic evolution of the sedimentary basins along with their stratigraphic history in the North Western Desert were made by the following authors^11,16^.

The majority of the petroleum fields in the Western Desert of Egypt including the Matruh Basin is associated with systems developed in the Late Cretaceous–Eocene and is located in or near the basin depocenters, which later became petroleum productive areas^17^. The Matruh Basin was initially developed during the Permian through Triassic times as a part of a large continental basin comprising the Shushan-Matruh Basin^29^. The Jurassic Tethyan rifting and drifting of the northern African plate towards the European plate resulted in the formation of the Matruh Basin along with other numerous rift basins^18,30^. Subsequently, at the end of Late Cretaceous, the collision of the African/Arabian and Eurasian plates produced compressive forces, which caused uplifting, folding, and basin inversion of large parts of the North Western Desert and formed the Syrian Arc System. These tectonic events resulted in the formation of faults and folds of NNE to SSW trends, which are dissected by normal faults of NW to SE trends (Fig. 1B). These fold systems are important petroleum structural traps for example in the Obaiyed, Jade, and Matruh fields^25^.

## Lithostratigraphy

The Matruh Basin has a sedimentary cover of nearly 14,000 ft (4267.2 m)^31^, which spans a wide range from the Cambrian to the Miocene (Fig. 2). The Matruh Basin has four main sedimentary cycles that controlled its sedimentation and are separated by regional unconformities^32,33^. The first cycle occurred throughout the Early to Middle Jurassic, which represents Ras Qattara and Yakout formation’s fluvial-lacustrine sediments and the Khatatba and Masajid formation’s deltaic and shallow marine sediments. The second cycle occurred in the latest Early to earliest Late Cretaceous (Albian–middle Cenomanian) and represents the fluvial-delta to shallow marine sediments of the following formations: Alam El-Bueib, Alamein, Dahab, Kharita, and Bahariya. The third cycle occurred in the Late Cretaceous (late Cenomanian–Campanian) and represents the Abu Roash open marine shale and the Khoman carbonates. The fourth cycle occurred during the late Paleogene and early Neogene periods (Eocene–Miocene) and represents the Apollonia, Dabaa, Moghra, and Marmarica formations open marine shale and carbonates^34,35^ (Fig. 2).

The Lower Cretaceous rock successions can be separated into four major formations of a shallow marine origin; these are the Alam El-Bueib, Alamein, Dahab, and Kharita^33^. The Alam El-Bueib Fm, which lies beneath the Alamein Dolomite and overlies the Masajid Formation, is mostly made up of sandstones, with minor amounts of siltstones, and shales with thin dominant limestone and dolomite, carbonate, beds. The thickness of the Alam El-Bueib Fm is large and contains six unconformable clastic rocks units, which were deposited above the Upper Jurassic marine carbonates of the Masajid Formation^34–38^.

## Materials and methods

### Materials

The data utilized in this study was obtained from Khalda Petroleum Company (KPC), with authorization granted by the Egyptian General Petroleum Corporation (EGPC). The dataset consists of well logs from four wells, namely Matruh 1-1, Matruh-5, Matruh-6, and Samaa-1X, which are distributed in the study area. These logs include “Gamma ray, Resistivity, Caliper, Neutron, Density, Photo Electric Factor, Density Environmental Correction, Acoustic logs, and Reservoir Pressure data. Reservoir pressure data (RFT) for three wells (Matruh-5, Matruh-6 and Samaa-1X) were available to be used in determining the reservoirs fluids behavior. Geochemical pyrolysis data (S_1_, S_2_, S_3_, Tmax, and PI) of 15 ditch samples from the AEB-6 Unit of the AEB Fm was available from the Matruh 1-1 well in the Matruh Oil Field (Table 1).

**Table 1:** Geochemical Pyrolysis data of source rock analyses for “15” ditch samples of Alam El Buieb Unit#6 (AEB-6), at Matruh-1-1 well, Matruh field, Western Desert (Provided from Khalda Petroleum Company, KPC).

S	Pyrolysis data matrouh 1-1 cutting samples of Alam El Buieb unit-6								Lithology			
	Depth	Depth	S1	S2	S3	Tmax	S2/S3	P.I	L.st	Shale	Sandstone	Coal
No	m	ft	mg/g	mg/g	mg/g	degC						
1	3626	11,896	0.29	0.5	1.39	452	0.35	0.37		20	80	
2	3660	12,008	0.28	0.43	1.71	447	0.25	0.4		40	60	
3	3702	12,146	0.53	1.33	1.83	437	0.72	0.28		60	40	
4	3714	12,185	0.3	0.66	2.35	446	0.28	0.31		70	30	
5	3756	12,323	0.2	0.92	1.88	448	0.48	0.23		50	50	
6	3774	12,382	0.39	0.94	1.76	447	0.53	0.3		60	40	
7	3806	12,487	0.42	0.78	2.24	451	0.34	0.35	30	30	40	
8	3840	12,598	0.46	0.93	1.95	448	0.47	0.33	20	60	20	
9	3868	12,690	0.37	0.73	1.8	451	0.4	0.24		40	60	
10	3894	12,776	0.22	0.45	1.92	439	0.23	0.33		90	10	
11	3934	12,907	0.54	0.88	1.67	449	0.52	0.38		60	40	
12	3958	12,986	0.6	1.01	2.24	450	0.45	0.37		70	30	
13	3988	13,084	0.86	2.74	1.83	453	1.49	0.24		40	55	5
14	4024	13,202	0.54	1.03	2.44	451	0.42	0.35		70	30	
15	4058	13,314	0.43	0.88	2.04	453	0.43	0.33		70	30	Tr

The provided data sets include also a two-dimensional survey of seismic reflection data (Fig. 1C) and a check-shot surveys in two wells (Matruh-5 and Matruh 1-1), which were used in constructing the structure depth map as an input for the reservoir modeling.

### Methodology

#### Source rock evaluation

The remaining hydrocarbon potential (S_2_) value was measured in mg HC/g rock, which represents the quantity of the hydrocarbons that can be produced via thermal cracking of the enclosed kerogen at a Tmax temperature range of (300–550 °C). It indicates the capability of the rock to generate hydrocarbons. S_2_ decreases with increasing maturity level giving false estimates of the source potential of the mature samples. Therefore, based on^38^ concept, the source generating potential in a mature rock should be corrected by adding the generated free HC (S_1_) to the remaining (S_2_) to give the original generating capacity (Genetic Potential: GP). The term used to denote the maximum temperature at which the highest rate of hydrocarbon generation (S_2_) occurs is called Tmax temperature, which is used as a maturity measure and it increases with increasing levels of maturity of organic matter. Caution should be taken with Tmax data, because the mineral matrix effect has the potential to suppress the Tmax values of bulk rock samples^38^.

#### Seismic interpretation

**Figure 3:**
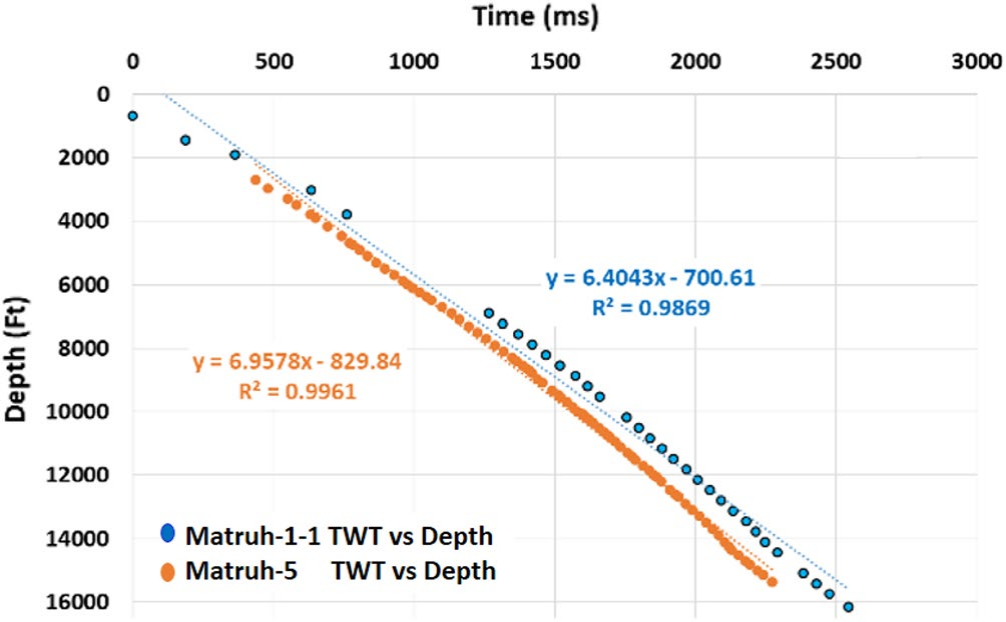
The time-depth relationship (TDR) between the Matruh 1-1 and Matruh-5 wells with a high R^2^ shows the confidence of the check-shot data used in the current study.

**Figure 4:**
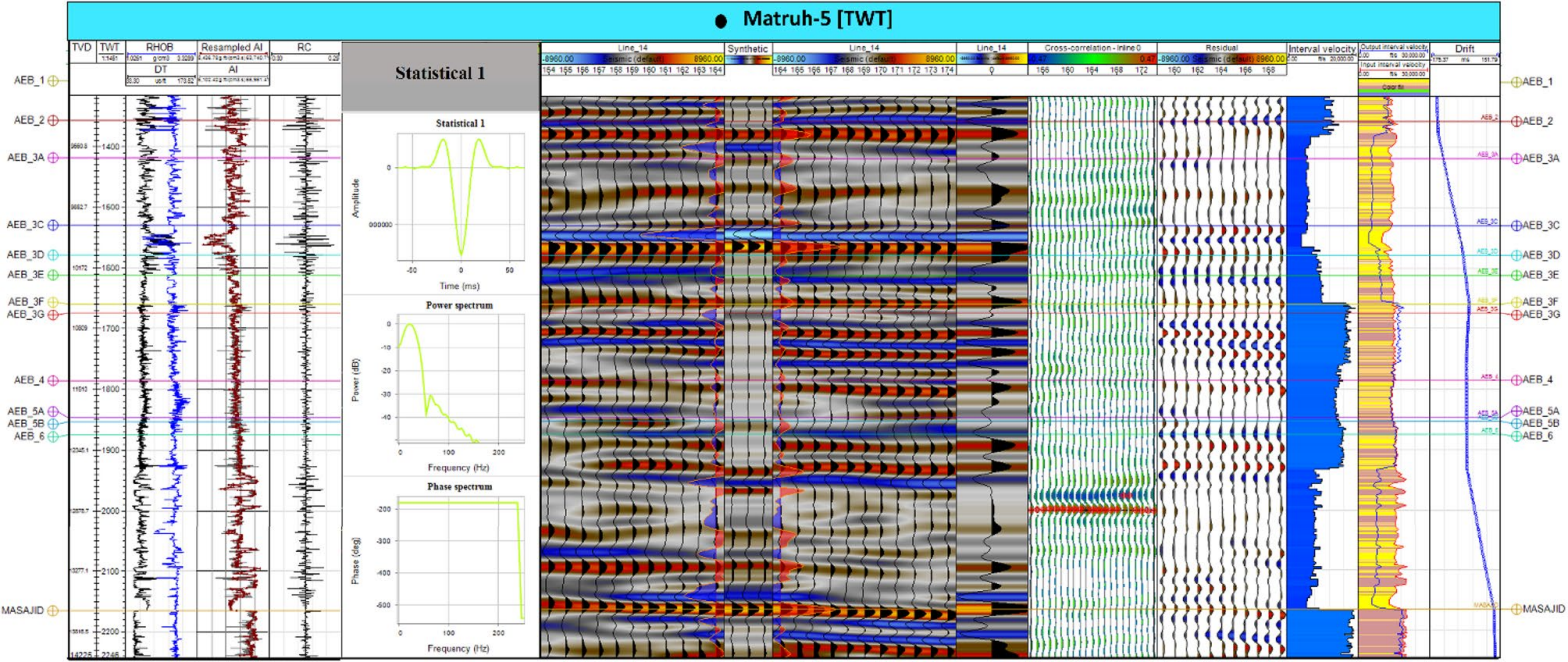
Synthetic seismogram at the Matruh-5 well, the Matruh Field at Matruh Basin (generated in Petrel ™ Schlumberger 2017.3 software, https://www.software.slb.com/).

**Figure 5:**
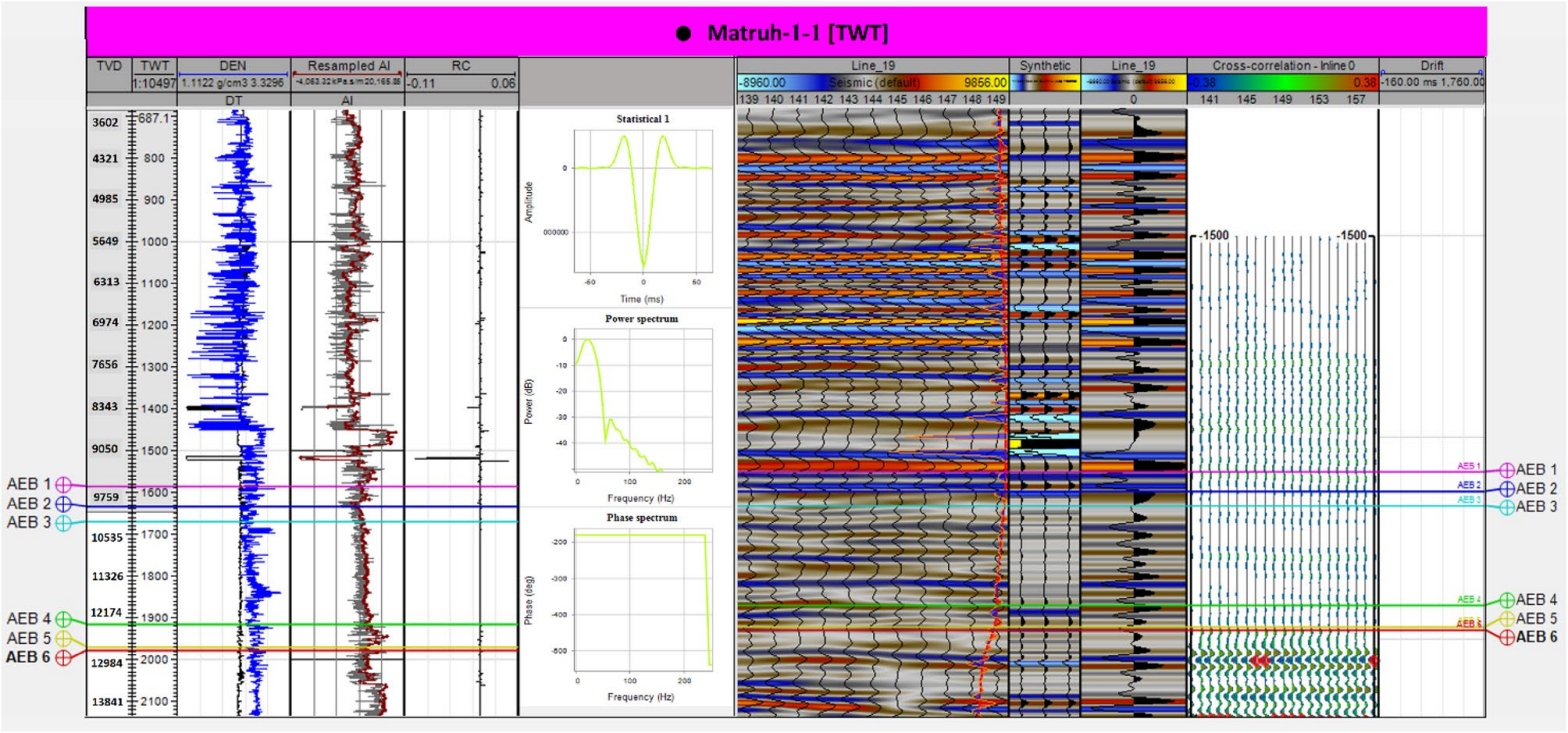
Synthetic seismogram at the location of Matruh-1-1 well, the Matruh Basin (generated in Petrel ™ Schlumberger 2017.3 software, https://www.software.slb.com/).

The seismic data given from the Khalda Petroleum Company (KPC) is in the form of 2D seismic data that has previously undergone shift correction, guaranteeing precise alignment. The ultimate goal of this work is to detect hydrocarbon accumulations, delineate their extent, and calculate their volumes. Therefore, seismic interpretation was based on picking and tracking laterally extended seismic reflectors for mapping geologic and stratigraphic features, and also for determining reservoir architecture^25,39–41^. Petrel software 2017.3 version was used in all geological and geophysical data integration to interpret the seismic data and to generate maps. The primarily step in the seismic interpretation was to apply the technique of seismic to well tie. The main purpose of this technique was to make a tie between seismic data (in time) and well data, formation tops, (in-depth) to find (seismic reflections). According to^36^, seismic data and geological features can be tied by applying two methods. Firstly, by creating a time-depth relationship using the check shot data (Fig. 3), which is a simple method but is less accurate. Secondly, by creating a synthetic seismogram (Fig. 4) using the formation velocity (i.e., sonic logs) and formation density (i.e., density logs) over the intervals of interest in the Matruh 1-1, and Matruh-5 wells. Before applying both methods, we made a quality control (Q.C.) on the available check shot data of the two wells on petrel and removed any outliers from them as presented in (Fig. 3). In the current study, we used the check shot survey of the Matruh 1-1 and Matruh-5 wells to connect the formation tops of the drilled wells in the field to the seismic section traveling through or near these wells (Figs. 4 and 5).

Any discrepancy between the generated synthetic seismogram and the seismic data were reduced by time-shifting methods used by^42,43^ or by changing in the wavelet phase constant used to construct the synthetic seismogram. Following the tie, picking horizons was based on tracking a reflection through a seismic line and correlating the intersections with the other lines to delineate the lateral extension for the analyzed layer, after interpretation. A Two-Way Time (TWT) maps were generated and used after depth conversion in generating structural maps that show the structural geology of the studied area^44^.

#### Petrophysical analysis

In the present studied domain, the petrophysical parameters of the sandstone units of the AEB Formation were estimated through the electrical well logs of four wells. The data utilized in this study includes the gamma-ray (GR), depth-resistivity (RT), density (ρb), neutron (ΦN), and sonic (Δt) logs in a digital format. Various types of well logs data were plotted and analyzed using Interactive Petrophysics (IP) version 2018 software.

##### Lithology interpretation

Interpreting the lithology of the target formation is generally gathered from core and cutting samples. However, we used the accessible well logging data (GR, ρb, ΦN, and Δt) to interpret the lithology of AEB Fm. After preparing and making Q.C. on the used logging curves, the first step of the petrophysical data analysis was to identify the lithological composition of reservoir units.

##### Shale volume (Vsh) estimation

High GR values reflect the presence of shale. Therefore, according to the used method by^45^, estimating the GR-Index, IGR is a mandatory to determine the shale volume (Vsh %) using the following Equ. (1):

1 $${\text{I GR}}\, = \,\left( {{\text{GR}}_{{{\text{log}}}} {-}{\text{GR}}_{{{\text{min}}}} } \right)/\left( {{\text{GR}}_{{{\text{max}}}} {-}{\text{GR}}_{{{\text{min}}}} } \right)$$

where: I _GR_ = Gamma ray index. GR _log_ = GR reading from log. GR _max_ = Maximum reading of GR in shale bed. GR _min_ = Minimum reading of GR in clean interval.

We calculated the Vsh% from the GR method using the following non-linear equation: Vsh = 0.33 (2^(2*I GR)^-1) used by^39,40^ for the early consolidated rocks (Equ. 1). In addition to the GR index method, the neutron-density combined logs method was used to assess the composition of shale. In this study, the Vsh was estimated using both methods. To obtain the most precise Vsh estimation, the procedure with the lowest Vsh was used. Along with the neuron and density logs, the estimated Vsh was applied to estimate the total and effective porosities.

##### Formation water resistivity (Rw)

**Figure 6:**
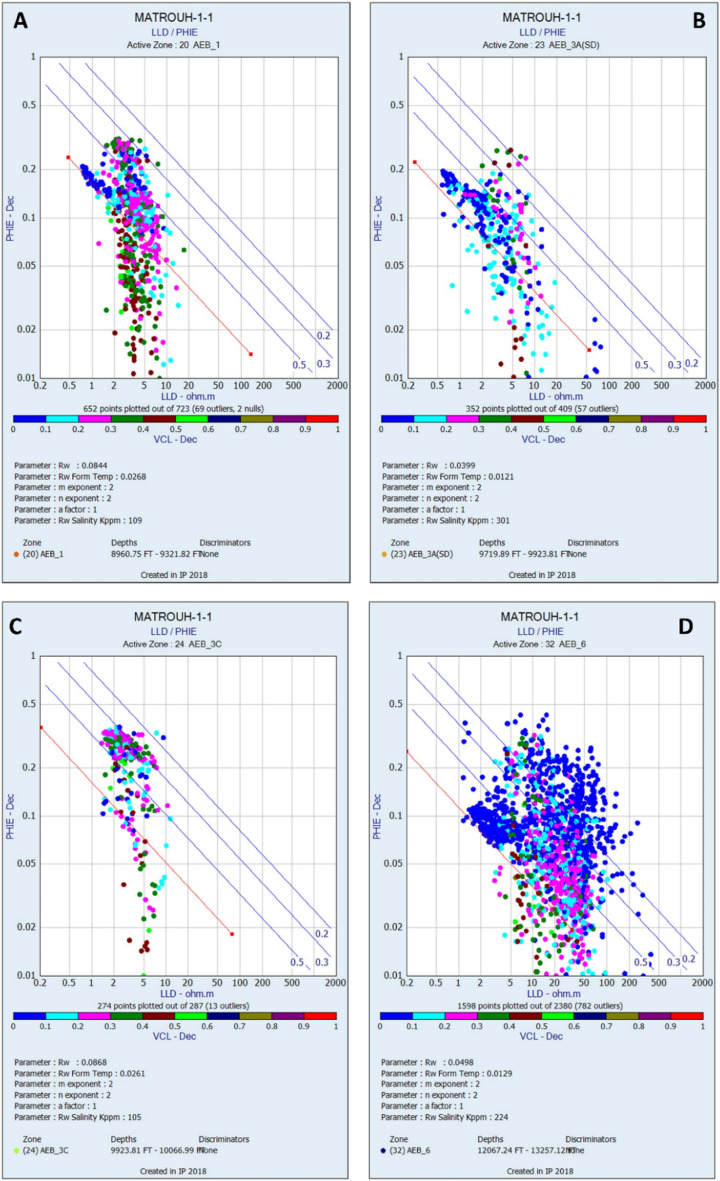
The Pickett’s plot for calculating the Alam El-Bueib Formation water resistivity (Rw) at the Matruh 1-1 well for members (**A**) AEB-1, (**B**) AEB-3A, (**C**) AEB-3C, and (**D**) AEB-6 (generated in IP = Interactive Petrophysics, 2018 software).

The resistivity log readings provide a record of the electrical conductivity of sedimentary deposits that contain a significant amount of clay minerals and indicate the presence of brines inside the pore spaces. In addition, porosity studies demonstrate a high level of sensitivity to the volume of fluid present inside geological formations. Consequently, the process of determining the resistivity of formation water (Rw) for the different units of the studied AEB Formation involved the use of resistivity and estimated porosity logs as illustrated by Picket’s plot (Fig. 6). The estimation of fluid saturation plays a crucial role in the assessment of hydrocarbon resources. To determine the formation water resistivity of the reservoirs, water-saturated zones were determined and employed to estimate the resistivity of water at various levels within the four chosen members of the Alam El-Bueib Formation. The AEB-1, AEB-3A, AEB-3C, and AEB-6 exhibit formation-water resistivity values of 0.084, 0.039, 0.086, and 0.049, respectively, as estimated by the Pickett’s plot methodology in the Matruh 1-1 well (Fig. 6). As a result, the fluid saturations were determined by using the estimated porosity, water resistivity, and resistivity logs.

#### Reservoir modeling

Geoscientists, geophysicists, and engineers can combine quantitatively all field data into updatable reservoir models using reservoir modeling, which is known as the “seismic simulation”. The fundamental purpose of reservoir modeling is to assess the hydrocarbon potential and give decision-makers with the valid data they require. The modeling of reservoirs incorporates geological and petrophysical modeling. Geological modeling covers the modeling of structures and facies, while petrophysical modeling deals with porosity and saturation modeling. The structural modeling depicts the region’s architecture and structural pattern. The facies model illustrates the vertical and horizontal distribution of the various lithology within the analyzed units. The area’s distribution of effective porosity and fluid saturation were shown via petrophysical modeling. In hydrocarbon exploration and production studies, the integration of all sorts of reservoir models is a powerful tool. Three-dimensional static reservoir modeling is also a useful technique for locating reservoir boundaries. Numerous logs from wells that have penetrated the investigated reservoirs were used to construct a facies model, which was followed by the construction of the property model (porosity and saturation) for the Matruh Field reservoirs^41–47^.

According to^47,48^, the process of creating a property model is composed of three consecutive phases: (1) geometric; (2) facies; and (3) petrophysical models. Creating a property model requires using all the available geological information to fill the grid cells of the structure model with petrophysical properties. Consequentially, data analysis and well logs scale-up will help in allocating well log data on the model 3D grid. Each grid cell will hold one value; therefore, averaging and scaling up of well log values were made to distribute the reservoir properties between the wells in the 3D-model.

##### Structural modeling

The first step in constructing the three-dimensional (3D) geological models is to create a structural model based on seismic data interpretation. Throughout the construction of the structural model, we used the formation tops acquired from the drilled wells in the research region, in conjunction with the depth-structure maps, to oversee and fine-tune the procedure. The basic goal of the 3D structural modeling is to understand the geological structure in three dimensions^49,50^. The key components required to develop a structural model are interpreting faults and horizons^40^. This framework of horizons and faults also serves as the geometrical input for building the 3D grid and can define the structural model of the AEB reservoirs. 20-2D seismic lines were used to pick the reservoir horizons, and to construct depth maps for all AEB Formation picked horizons. The AEB Fm was divided into zones from the well logs formation tops that prepared based on the tie with the picked horizons. The thickness of each layer designates the number of cells present in each zone, which are then subdivided into layers of equal thickness. Before moving to the next phase of facies modeling process, much consideration should be taken into layering the zones of interest through upscaling of well logs.

##### Facies modeling

Facies analysis should be applied to understand the sedimentology of the formation through performing high-resolution stratigraphic correlations^51–53^. Facies modeling techniques in petrel software are separated into stochastic, deterministic, and interactive methods. Stochastic model technique was regularly used when the available data are little. As a first step in proceeding to facies modeling, facies logs must be created, followed by the upscaling of these logs to the geological grid^53^. Through an equation constructed using Petrel software, the gamma ray logs were used to generate discrete facies logs. As the second stage in the facies modelling process, a scaling up of the created reservoir facies log in the 3D-model was made^54^.

##### Petrophysical modeling

The purpose of petrophysical modeling is to incorporate fine-scale reservoir porosity and fluid saturation into a 3D grid model. For petrophysical modeling, scaling-up log-derived petrophysical parameters are used to build models. These models were then distributed in the area specified by the facies distribution. During the process of static reservoir modeling, it was extremely important to distribute these features, whether they are continuous or discrete over the grid by assigning a value to each cell in the grid. Using the geophysical logs that were provided for the given wells in the research area, the effective porosity and oil saturation were estimated, and their values were scaled up and dispersed on the wells. The features that were used in constructing the effective porosity and oil saturation models were guided by the structural and facies models^55–60^.

## Results

### Source rock evaluation

**Figure 7:**
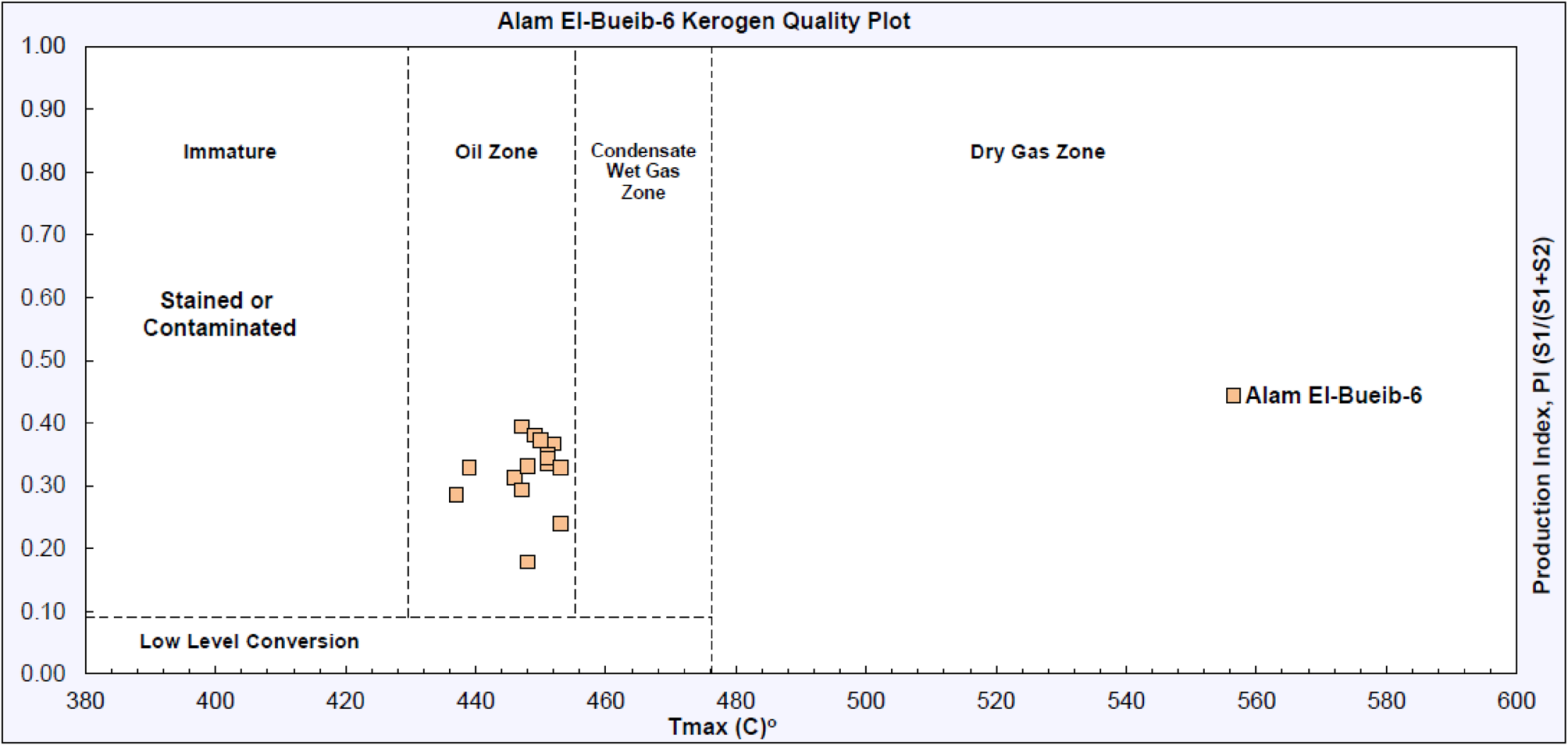
The kerogen quality plot of AEB-6 (SH) shale intervals shows the 15 examined samples are located in the oil-window zone.

The 15 samples recovered from the shale intervals of the AEB-6 member in the Matruh 1-1 well were analyzed by Rock–Eval pyrolysis. All 15 samples are mature, where they are located in the oil-window zone (Fig. 7, Table 1). The relatively high production indices (PI: 0.24–0.39) are consistent with the Tmax values and further support the interpreted peak oil-window for the Alam El-Bueib Formation (Fig. 7).

**Figure 8:**
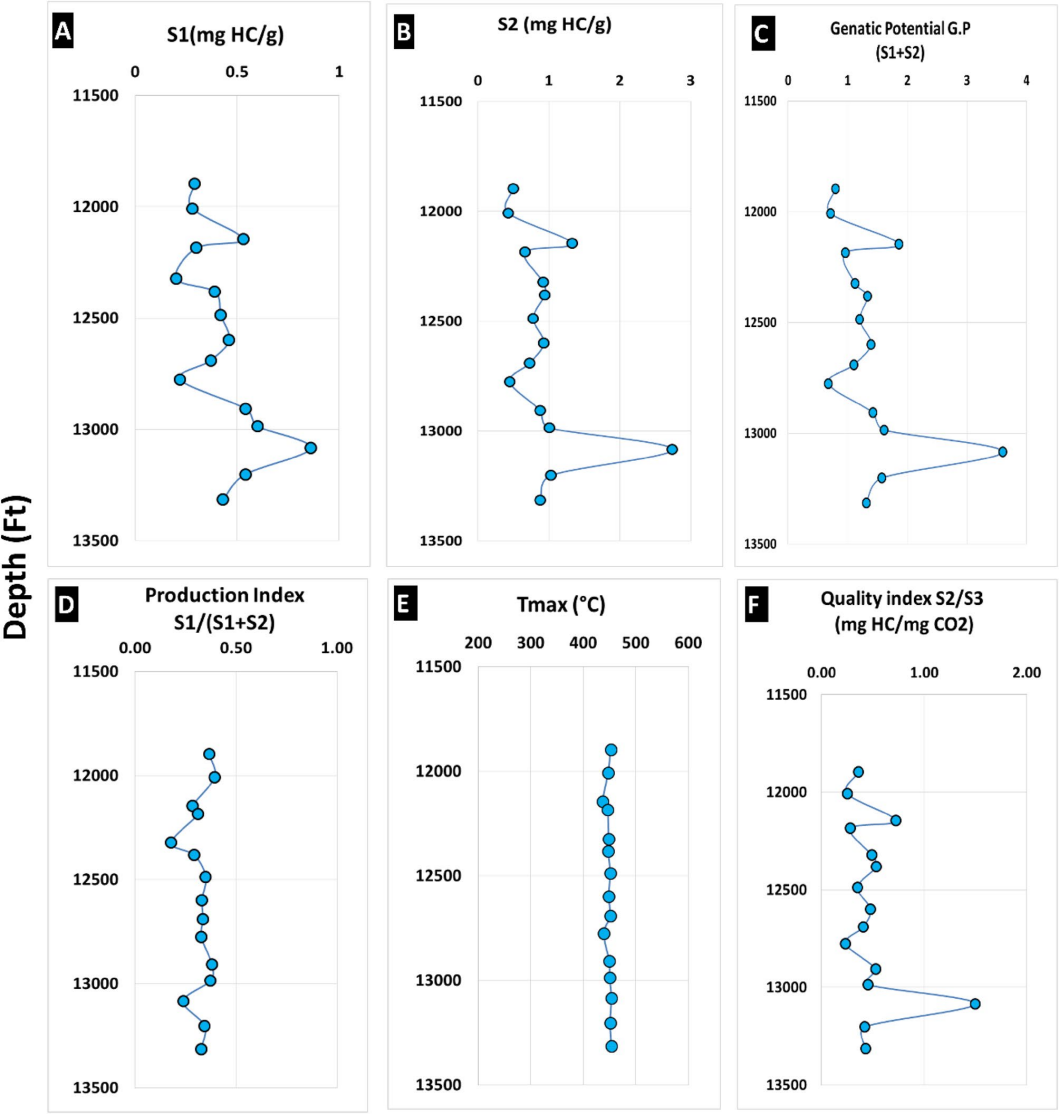
Rock–Eval pyrolysis data at the Matruh 1-1 well for the Alam El-Buieb unit-6 (AEB-6), the Matruh Oil Field. (**A**) S1: The generated free hydrocarbons (H.C.), (**B**) Remaining hydrocarbon potential (S2), (**C**) Genetic potential (GP: S1 + S2), (**D**) Production index (IP: S1/S1 + S2), (**E**) Tmax, (**F**) Hydrocarbon quality index (S2/S3).

For the Unit-6 of Alam El Bueib Formation samples, S1 values (Fig. 8A) which represents The hydrocarbons that undergo vaporization and expulsion from the sample at low temperatures (~ 300 °C) and are quantified in mg HC/g rock vary between 0.2 and 0.86 mg HC/g rock (average 0.43 mg HC/g rock). The pyrolysis-derived (S2) values vary between 0.43 and 2.74 mg HC/g rock (average 0.95 mg HC/g rock) (Fig. 8B) represents the amount of hydrocarbons generated through thermal cracking of the contained kerogen at temperature (~ 300–550 °C). It indicates the capability of the rock to generate hydrocarbons and represents the remaining generative capacity of the rock, which decreases with increasing maturity level, giving a false estimate of the source potential of the mature samples. Therefore, the source generating potential in a mature rock should be corrected by adding the generated free (S1) to the remaining (S2) to give the original generating capacity^59^. S3 values are between 1.39 and 2.44 mg CO2/g rock (average 1.94 mg CO2/g rock) (Table. 1). Overall, the shale interval of the AEB-6 member is characterized by low S_1_ values and low S_2_ values, which indicate poor oil-generating potential. However, the coaly shale Sample 13 at depth (13,084 ft/3988 m) shows a relatively high S_2_ value (2.74 mg HC/g rock) and high genetic potential (GP = S1 + S2) value (3.6 mg HC/g rock) (Fig. 8C) due to its higher organic richness^21,23^. The sum of S1 and S2 that represents the genetic potential (G.P.) of the source rock (Fig. 8C) refers to the maximum quantity of oil and gas that a specific amount of the rock may generate under ideal conditions of burial depth and duration^23^. While the Production Index (PI), expressed as S1/(S1 + S2) (Fig. 8D), serves as the transformation ratio and provides insight into the degree of thermal maturation as well as the existence of migrating hydrocarbons^7^, the PI values for Unit-6 of the Alam El Bueib Formation vary between 0.23 and 0.4, with an average of 0.32. Furthermore, the consistency of the Tmax values (Fig. 8E) with depth across the Matruh 1-1 well indicates the occurrence of indigenous oil in the AEB-6 member, as contamination with migrated oil is characterized by suppressed Tmax values and abnormal S_1_ values, which are not detected here^7,61^^.^ The hydrocarbon quality index (S2/S3) of AEB-Unit-6 (Fig. 8F) varies between 0.23 and 1.49 mg HC/mg CO_2_ with an average of 0.49 mg HC/mg CO_2_.

### Reservoir petrophysics

**Figure 9:**
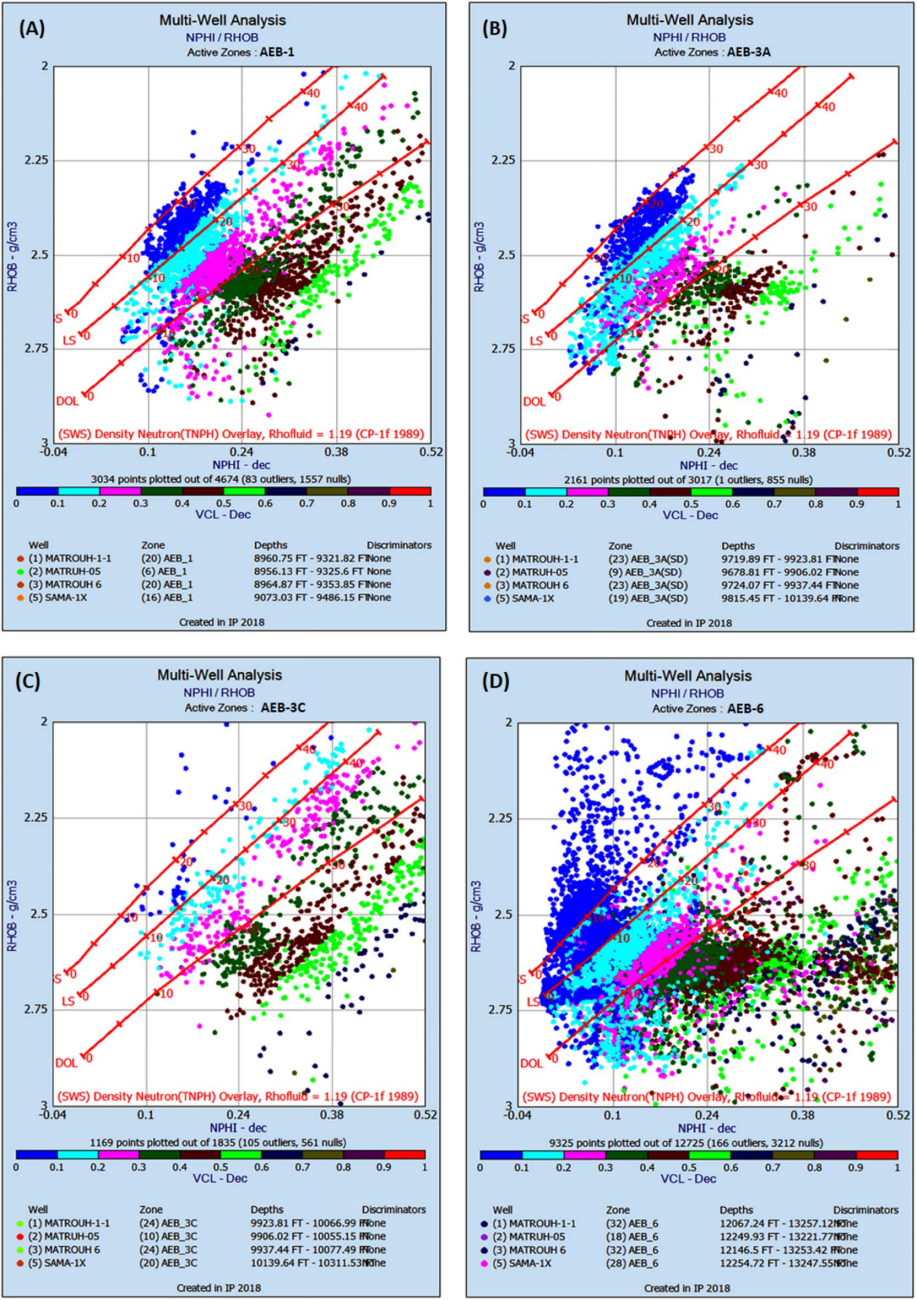
The Neutron-Density cross plots showing the lithological content of: (**A**) AEB-1, (**B**) AEB-3A, (**C**) AEB-3C, and (**D**) AEB-6 (Generated in IP = Interactive Petrophysics, 2018 software).

The lithology content of AEB reservoirs was determined using log responses and log-derived cross plots. The neutron-density cross plots of the IP software were used to estimate the lithology of these members, as illustrated in Fig. 9. Four out of seven reservoir intervals of the Alam El-Bueib Formation (AEB-1, AEB-3A, AEB-3C, and AEB-6) were selected for evaluation. Because their assessed petrophysical characteristics show interesting results along the four wells in the current study; these intervals are distinguished by shale and sand intervals. The litho-saturation crossplot known in the oil industry as Computational Petrophysical Interpretation (CPI) is widely used as a vital and reliable tool for deciding whether to complete, reject, or set aside some formations for possible future testing.

**Figure 10:**
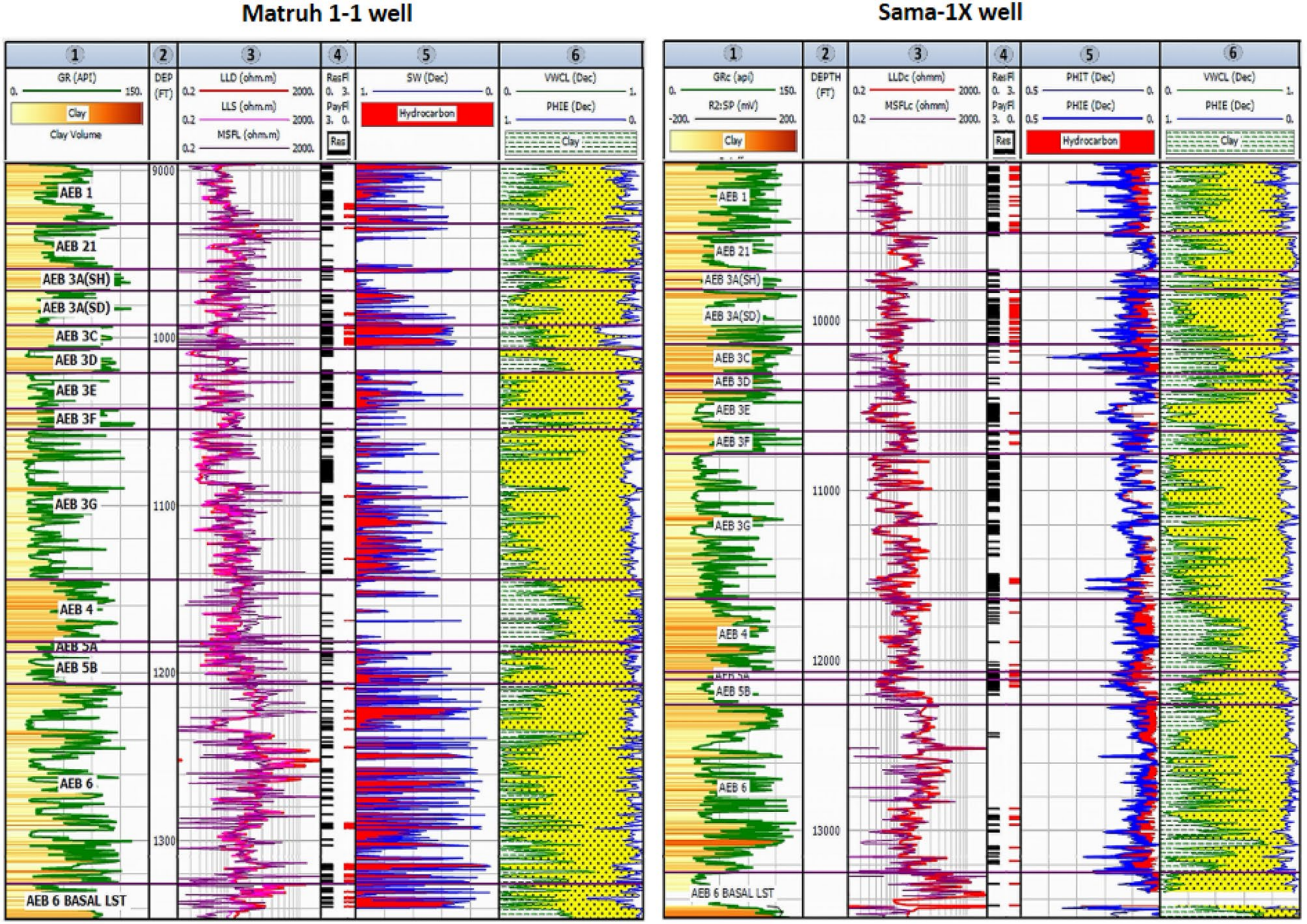
The lithosaturation panel of the Alam El-Buieb Formation for the Matruh-1 well (left) and Sama-1X well (Right) (Generated in IP = Interactive Petrophysics, 2018 software).

**Figure 11:**
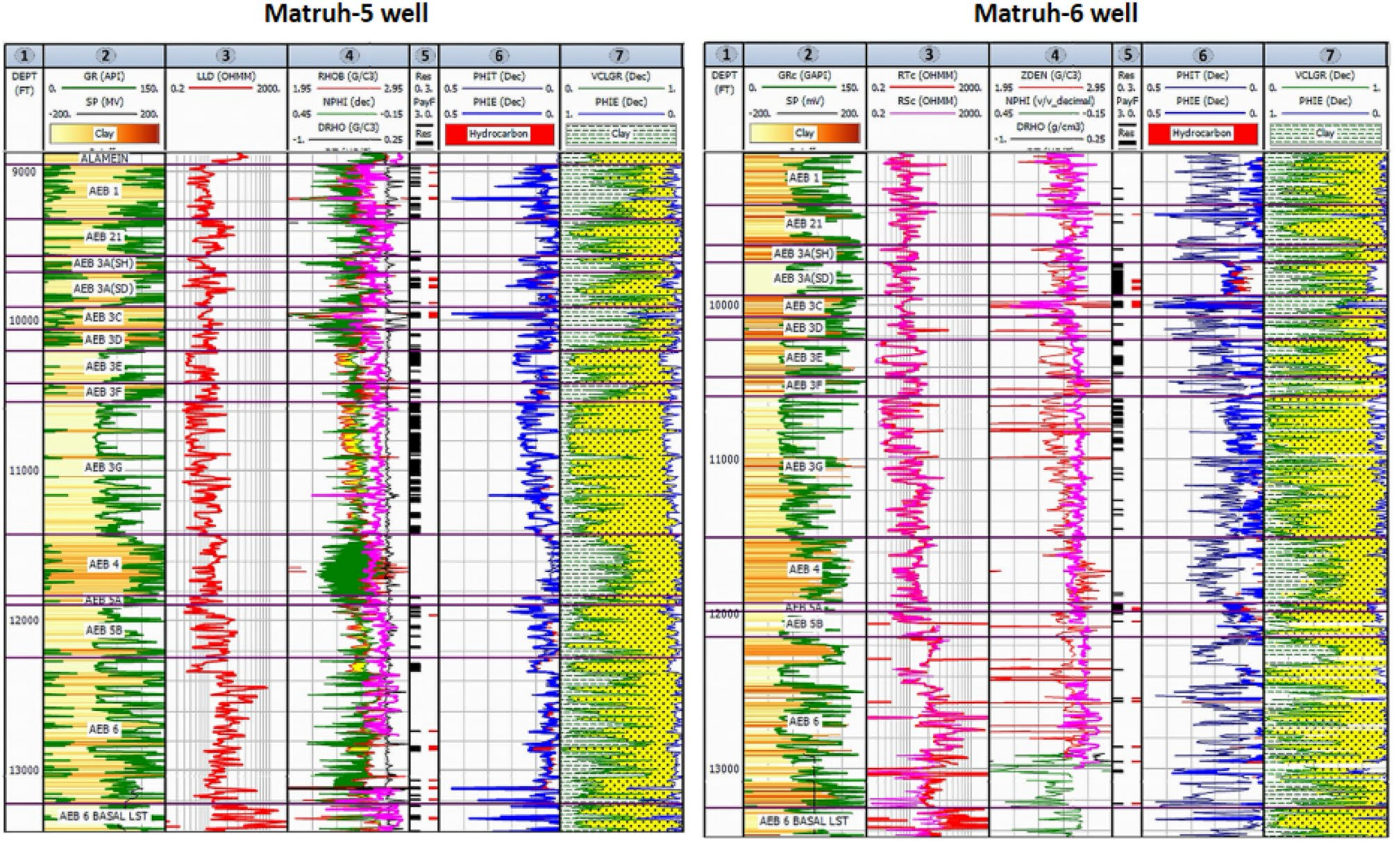
The lithosaturation panel of Alam El-Buieb Formation for the Matruh-5 well (left) and Matruh-6 well (Right) (Generated in IP = Interactive Petrophysics, 2018 software).

The litho-saturation crossplots of AEB Fm for the four wells encompassed many parameters such as shale volume (Vsh%), effective porosity (Φeff), net pay, gross thickness, and hydrocarbon saturation. These parameters were computed and visually shown in Figs. 10 and 11. In addition, the computed petrophysical parameters for the wells in the Matruh Field were compiled and shown in Table 2. As we can see in track 3 (Figs. 10 and 11), the litho-sturation panel of the four analyzed wells indicates that there is a continuous net pay flag for the selected four units of the AEB Fm. While the net pay flag in the other members of the Alam El-Bueib Formation, AEB-3E, AEB-4, and AEB-5, is not continuous in all wells. This justifies why we based our whole-work analysis on the selected four units.

**Table 2:** The average values of the petrophysical parameters calculated for the studied members of the AEB Formation (calculation have been made in IP, 2018 Software).

Well name	Member	Net pay thickness (ft)	Vsh (%)	Effective porosity (%)	Oil saturation (%)
Matruh-1	AEB-1	37	18	19	56
	AEB-3A	18	17	14	55
	AEB-3C	73	22	27	64
	AEB-6	160	10	19	80
Matruh-5	AEB-1	24	20	27	73
	AEB-3A	38	5	15	53
	AEB-3C	24	22	27	80
	AEB-6	68	5	20	81
Matruh-6	AEB-1	13	6	27	73
	AEB-3A	48	5	16	52
	AEB-3C	16	12	30	85
	AEB-6	20	5	17	88
Sama-1x	AEB-1	112	14	15	61
	AEB-3A	139	12	14	66
	AEB-3C	19	14	13	75
	AEB-6	35	20	14	70

Results show the average petrophysical calculations of the selected four AEB units (Table 2). Firstly, the AEB-1 member has an average net pay thickness, which ranges between a lowest value of 13 feet in the Matruh-6 well and a highest value of 112 feet in the Sama-1X well. The average percentage of shale volume (Vsh%) varies between 6% at the Matruh-6 well and 20% at the Matruh-5 well. The effective porosity ranges between 15% at the Sama-1X-well and 27% at the Matruh-5 and Matruh-6 wells. The hydrocarbon saturation ranges between 56% at the Matruh 1-1 well and 73% at the Matruh-5 and Matruh-6 wells. Secondly, the petrophysical evaluation of the AEB-3A member shows an average net pay thickness, which ranges between 18 and 139 feet in the Matruh 1-1 and the Sama-1X wells, respectively. Vsh% ranges between 5 and 17% in the Matruh-5 and Matruh 1-1 wells, respectively.

The effective porosity ranges between 14 and 16% in the Matruh 1-1 and Matruh-6 wells, and the hydrocarbon saturation ranges between 52 and 66% in the Matruh-6 and Sama-1X wells. Thirdly, the average net pay thickness of the AEB-3C Unit is 16–73 feet in the Matruh-6 and Matruh-1 wells, and the average shale volume is 12% in the Matruh-6 and 22% in the Matruh-1 and Matruh-5 wells. In addition, the average effective porosity is 13% in the Sama-1X well and 30% in the Matruh-6 well, and the hydrocarbon saturation is 64% in the Matruh-1-1 well and 85% in the Matruh-6 well. Finally, the AEB-6 member has an average net pay of 20 feet in the Matruh-6 well and 160 feet in the Matruh 1-1 well, a Vsh% range of 5–20% in the Matruh-5 and Sama-1X wells, an average effective porosity range between 14–20% in the Sama-1X and Matruh-5 wells, and a hydrocarbon saturation range of 70–88% in the Sama-1X and Matruh-6 wells, respectively.

### Seismic interpretation

**Figure 12:**
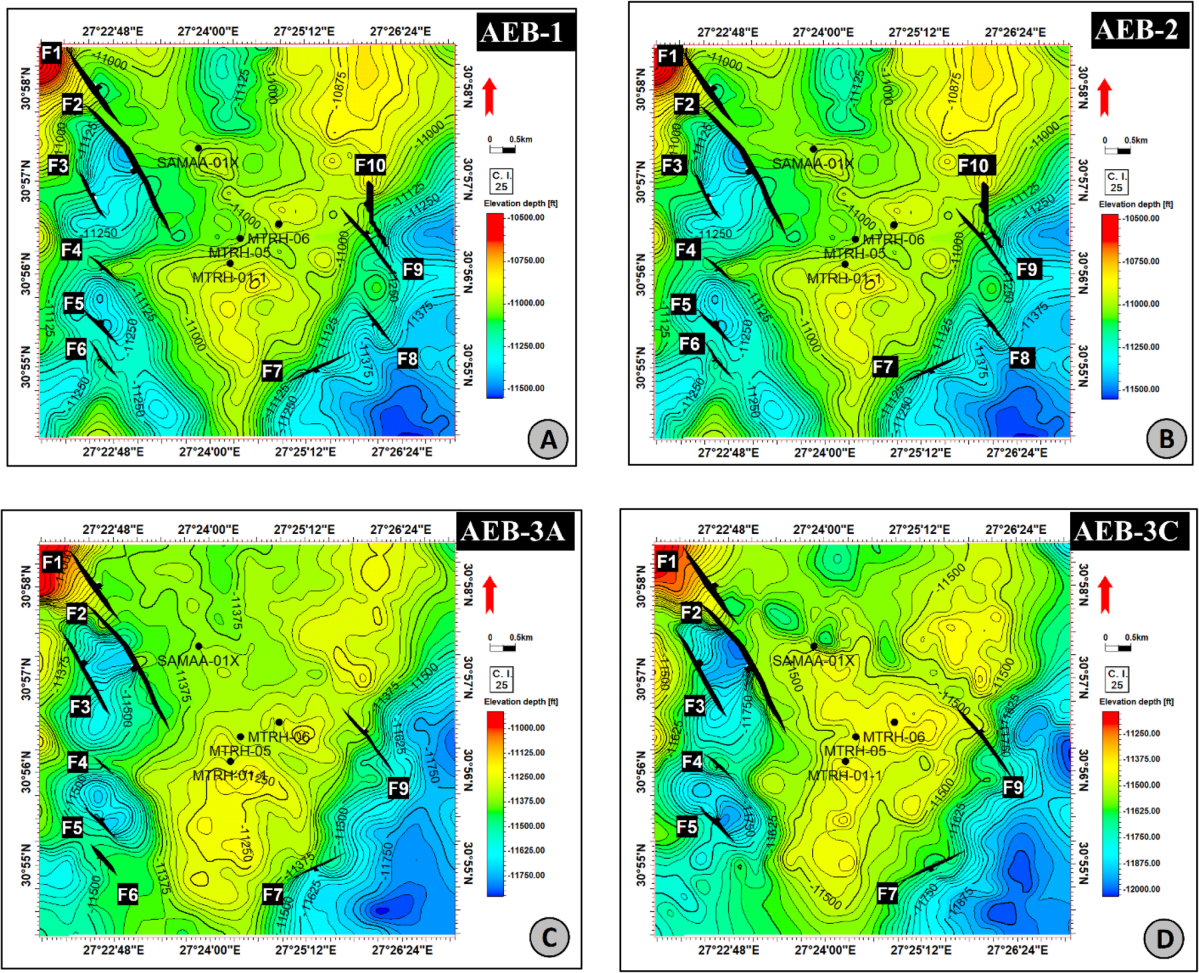
Depth structure contour map on the top of (**A**): AEB-1 member; (**B**) AEB-2 member; (**C**) AEB-3A member; (**D**) AEB-3C member. (Maps generated in Petrel ™ Schlumberger 2017.3 software, https://www.software.slb.com/products/petrel).

**Figure 13:**
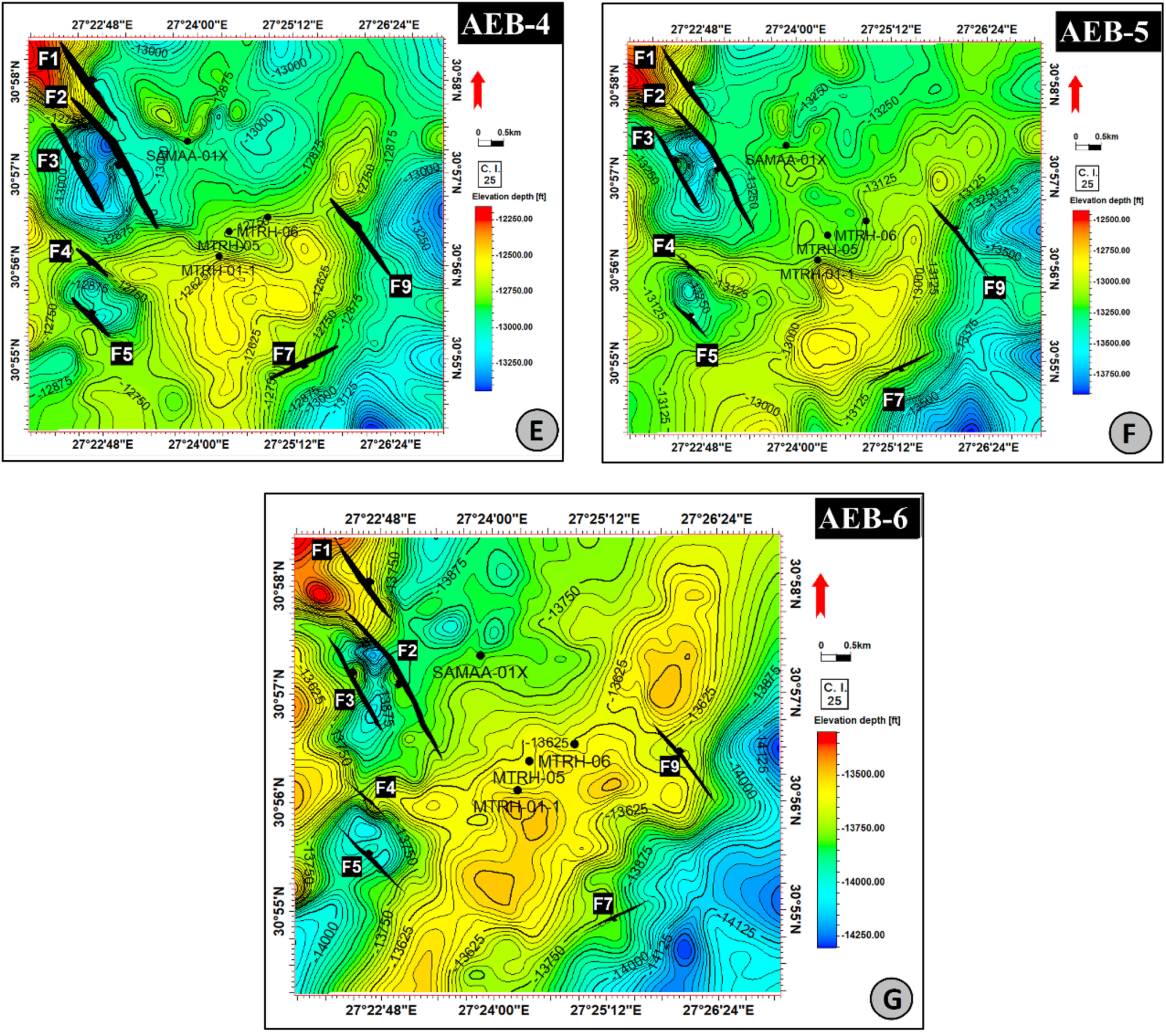
Depth structure contour map on the top of (**A**) AEB-4 member; (**B**) AEB-5 member; (**C**) AEB-6 member. (Maps generated in Petrel ™ Schlumberger 2017.3 software, https://www.software.slb.com/products/petrel).

Depth conversion is a key technique next to interpretation because seismic data are measured in the time domain, while well data is measured in the depth domain. So, depth conversion was made for all-time structure maps, which were later used in mapping target horizons to define the reservoir volumes of certain hydrocarbon accumulations. In the present study, members of the AEB Fm were tracked in all available seismic sections, and TWT contour maps and structure depth conversion maps were constructed for them (Figs. 12 and 13).

**Figure 14:**
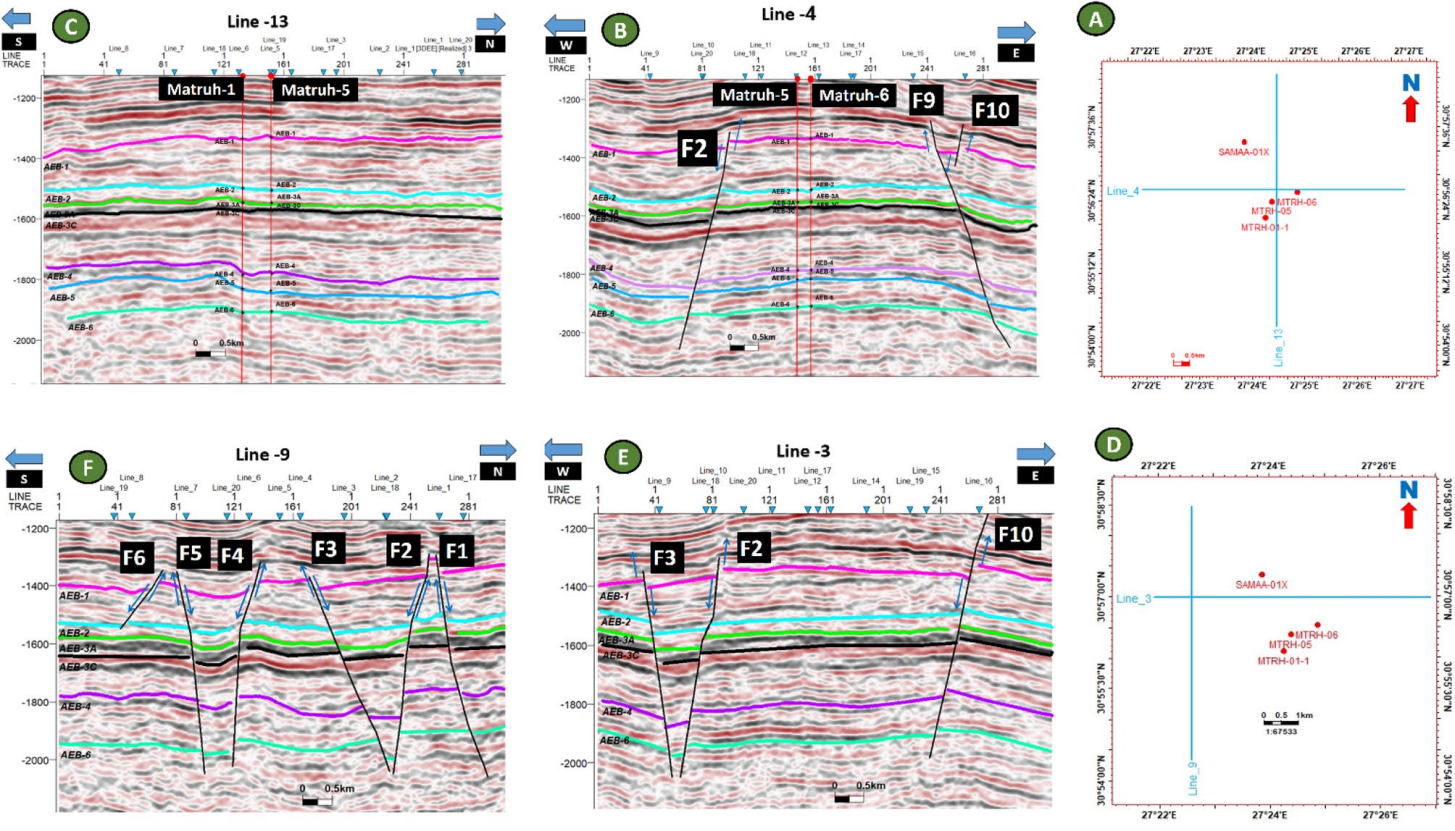
Four interpreted seismic sections showing the representative faults affecting the Alam El-Buieb Formation: (**A**) Index map of the two interpreted Line-4 (E-W) and Line 13 (S–N) seismic sections (**B**) Seismic line 4 passes through Matruh-5 and Matruh-6 wells. (**C**) Seismic line 13 passes through Matruh-1-1 and Matruh-5 wells. (**D**) Index map of the two interpreted Line-3 (E-W) and Line 9 (S–N) seismic sections. (**E**) Seismic Line-3 in the (W-E) direction is cut by three normal faults. (**F**) Seismic line 9 in the S–N direction, cut by a group of normal faults, makes a horst and grabens on the top of the Alam El-Buieb formation.

**Figure 15:**
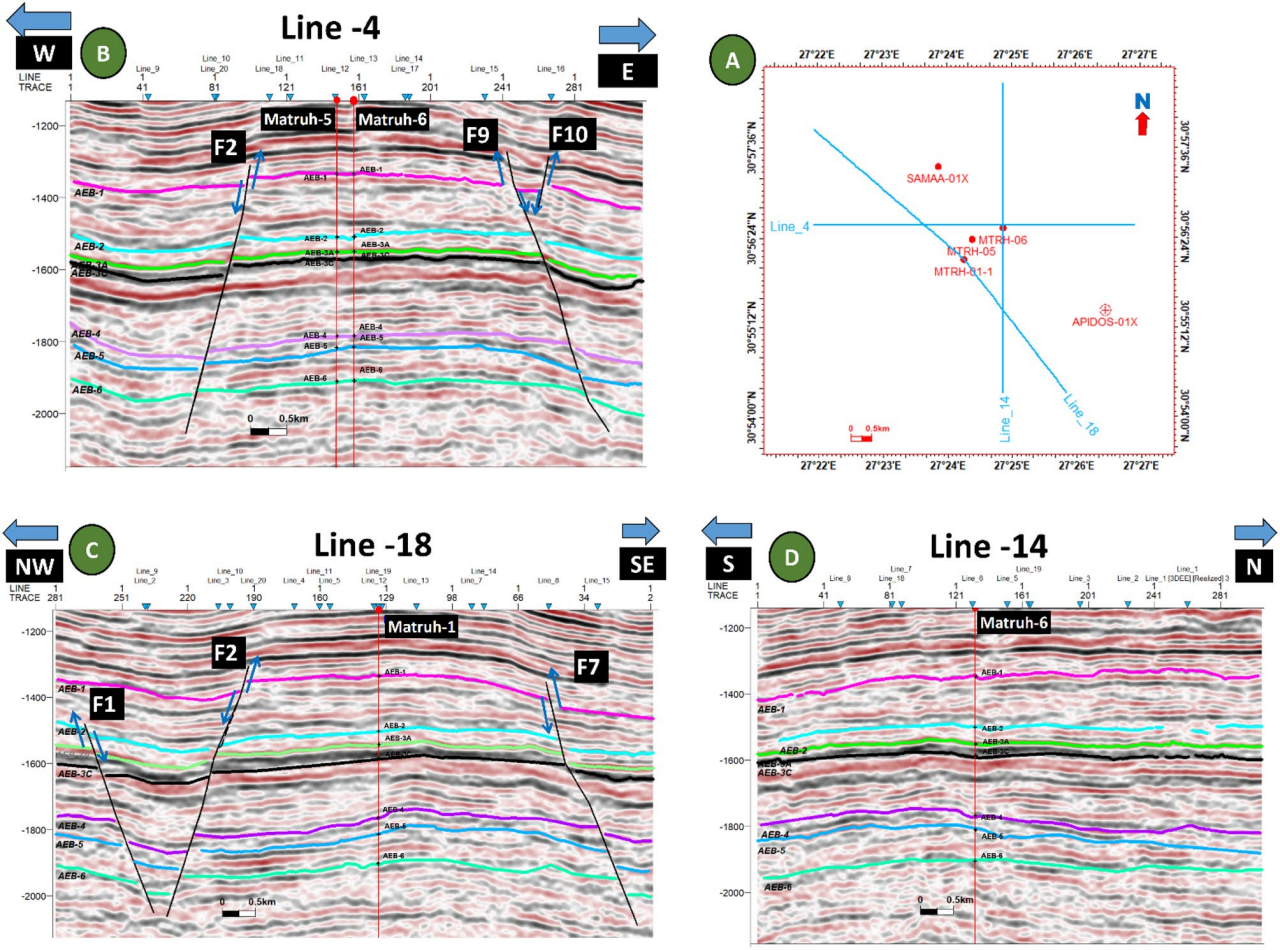
Two interpreted seismic sections showing the representative faults affecting the Alam El-Buieb Formation: (**A**) Index map of three interpreted seismic sections showing the faults affecting the Alam El-Buieb Formation: (**B**) Line 4 in the (E-W) direction, passing through Matruh-5 and Matruh-6 wells. (**C**) Line 14 in the (S–N) direction, passing through Matruh-6 well. (**D**) Line 18 in the NW–SE direction, passing through Matruh-1-1 well.

According to the analysis of the seismic data, the studied region is affected by around of ten normal faults. Certain faults impact every level of the AEB Formation, whereas others do not. A total of ten normal faults (F1 to F10) as seen in the depth structure contour map constructed on the top of the AEB Formation’s members (Figs. 12 and 13). These faults affect both AEB-1 and AEB-2 units (Figs. 12A and B). A total of eight normal faults, labeled F1 to F7 and F9 in Fig. 12C, have an effect on AEB-3A. Seven normal faults (F1 to F5, F7, and F9), as seen in Figs. 12D, E, F, and G, impact AEB-3C, AEB-4, AEB-5, and AEB-6 units, respectively. All observed faults exhibit a NW–SE orientation that aligns with the trend of the Gulf of Suez, with the exception of F7, which follows the NE-SW Aqaba trend. The middle section denotes the most elevated location within the mapped members of the AEB Fm in the region. The constructed depth-structure maps describe fault types that are found on the examined surfaces of the AEB Fm. Many representative faults were interpreted, some of which are shown in Figs. 14 and 15.

The interpreted faults and horizons were also used to make time-structure contour maps for the seven members of the AEB Formation. By connecting the formation tops from well logging data to 2D seismic lines, the main units of the AEB Formation could be identified. We used the available check shot survey to calibrate the sonic log for the studied wells in the current study. Then, acoustic impedance was determined using density and calibrated sonic logs, which were later used to calculate the reflectivity coefficient. Synthetic seismograms were made for the selected horizons. Then, velocity information was used later to create time-depth conversion maps, which were converted to a structure depth map that was used later to construct the 3D-structure model.

### Structure model

**Figure 16:**
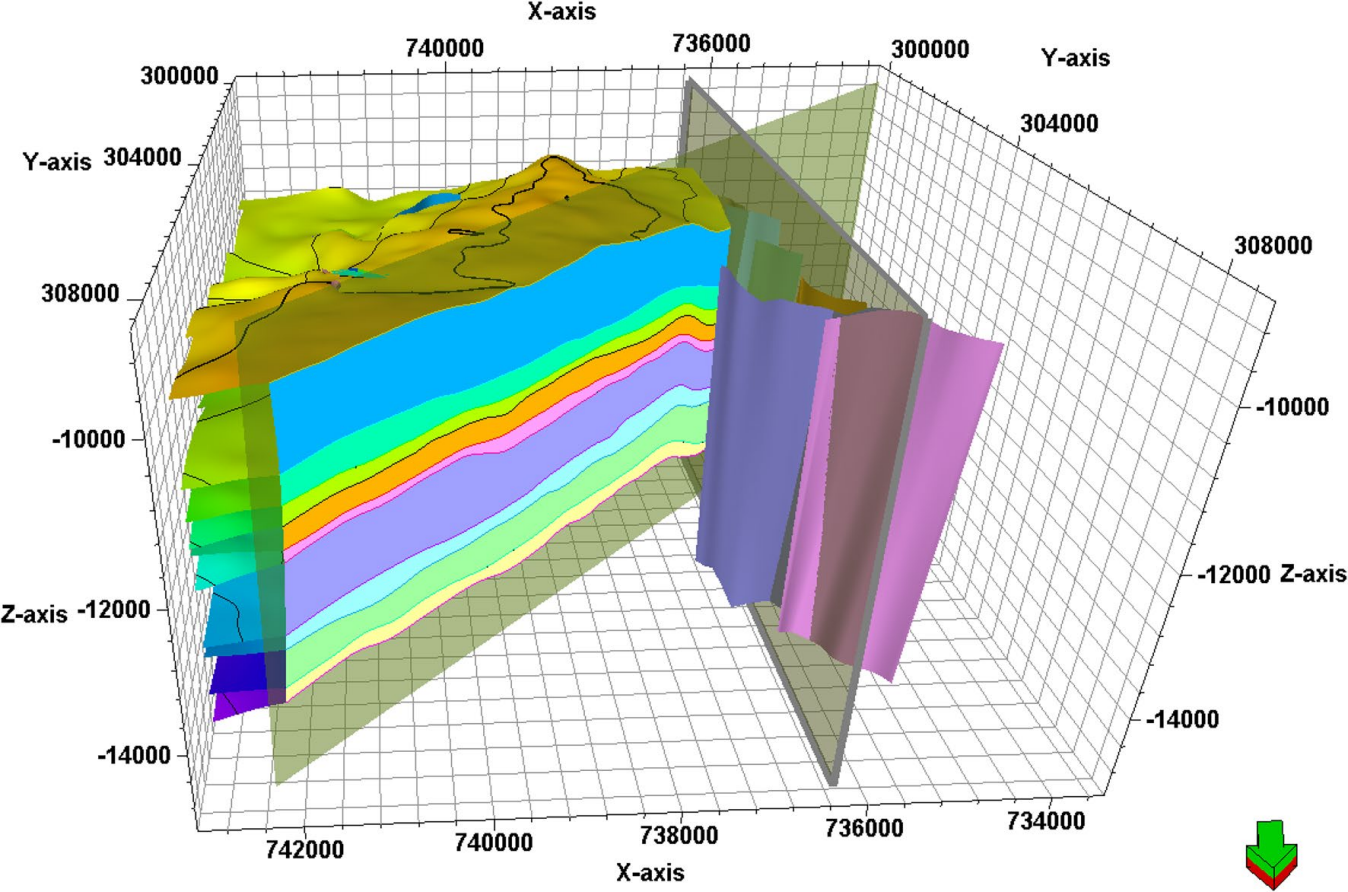
The 3D-structure model of Alam El-Bueib Formation in the Matruh Field.

The main purpose of generating a model containing lithology, petrophysical parameters, and structure is to estimate the geology and evaluate the target formations in the undrilled locations^58^. Consequently, the provided seismic data helped in defining the structural patterns that affect the AEB Fm in the location of the studied wells. The constructed 3D model of Alam El-Bueib Formation units (Fig. 16) mostly comprises the structural framework of the Matruh Basin.

The boundary of the Matruh Field was delineated by interpreting the fault sticks from the 2D seismic survey to build a fault model in the time domain, which was later converted into the depth domain through the velocity model. The availability of time-depth data helped in the creation of TWT structure maps and structure-depth maps for the Alam El Buieb Formation unites, as shown in Figs. 12 and 13. This represents the converted structure depth contour map from the TWT structure maps of the seven members of the Alam El-Bueib Formation. The developed structure maps for the AEB Formation’s members demonstrate that the structures that affect the area have two main trends: NW–SE and NE-SW. These maps were used to create the structural model in Fig. 16.

**Figure.17:**
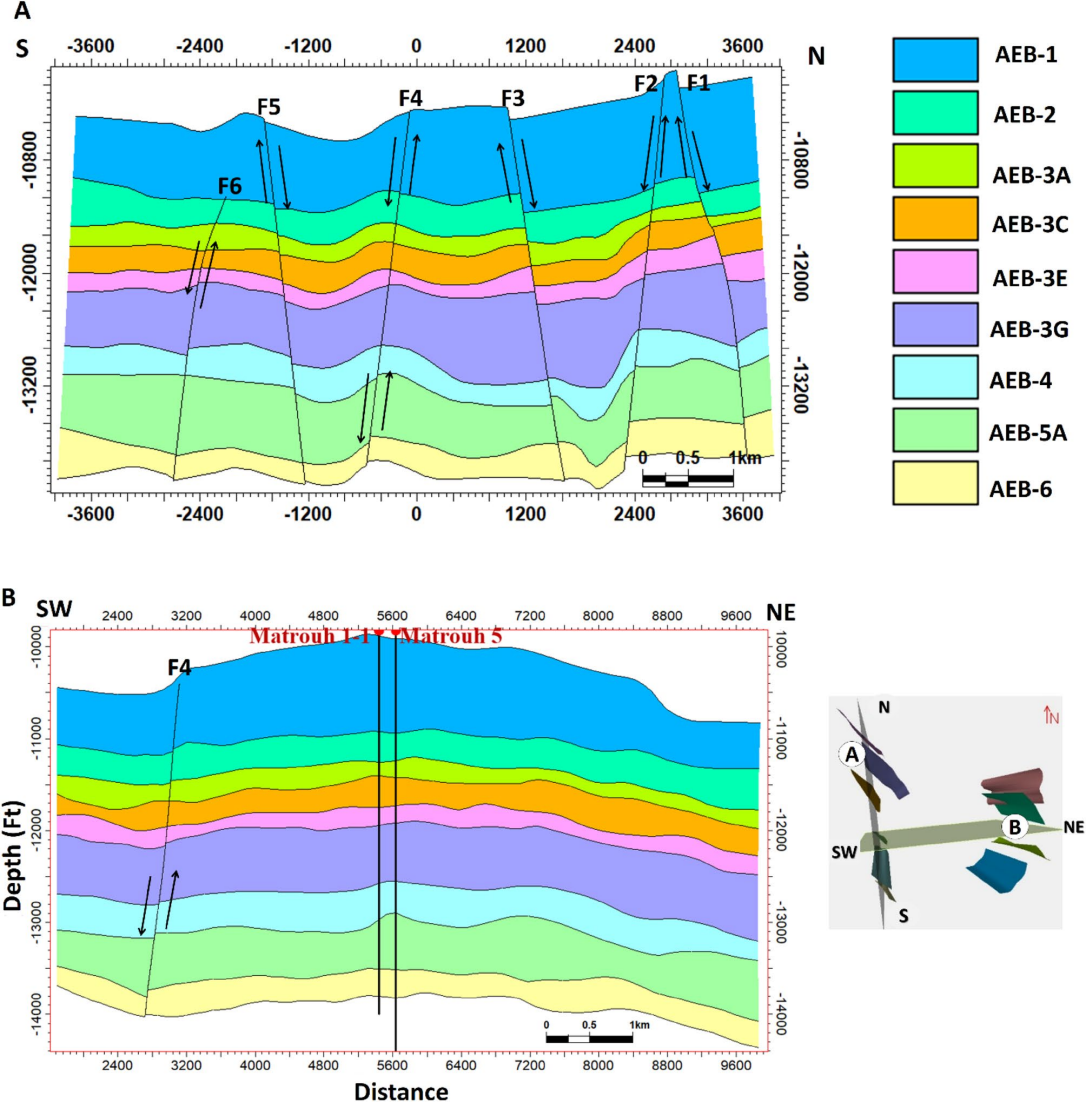
Two intersections through the 3D structural model of the Alam El-Bueib Formation members are in the direction of South-North as in (**A**) and in the direction of SW-NE as in (**B**). F1-F6 are the number of faults. Arrows on the figure indicate the up- and down-thrown sides of the faults.

**Figure 18:**
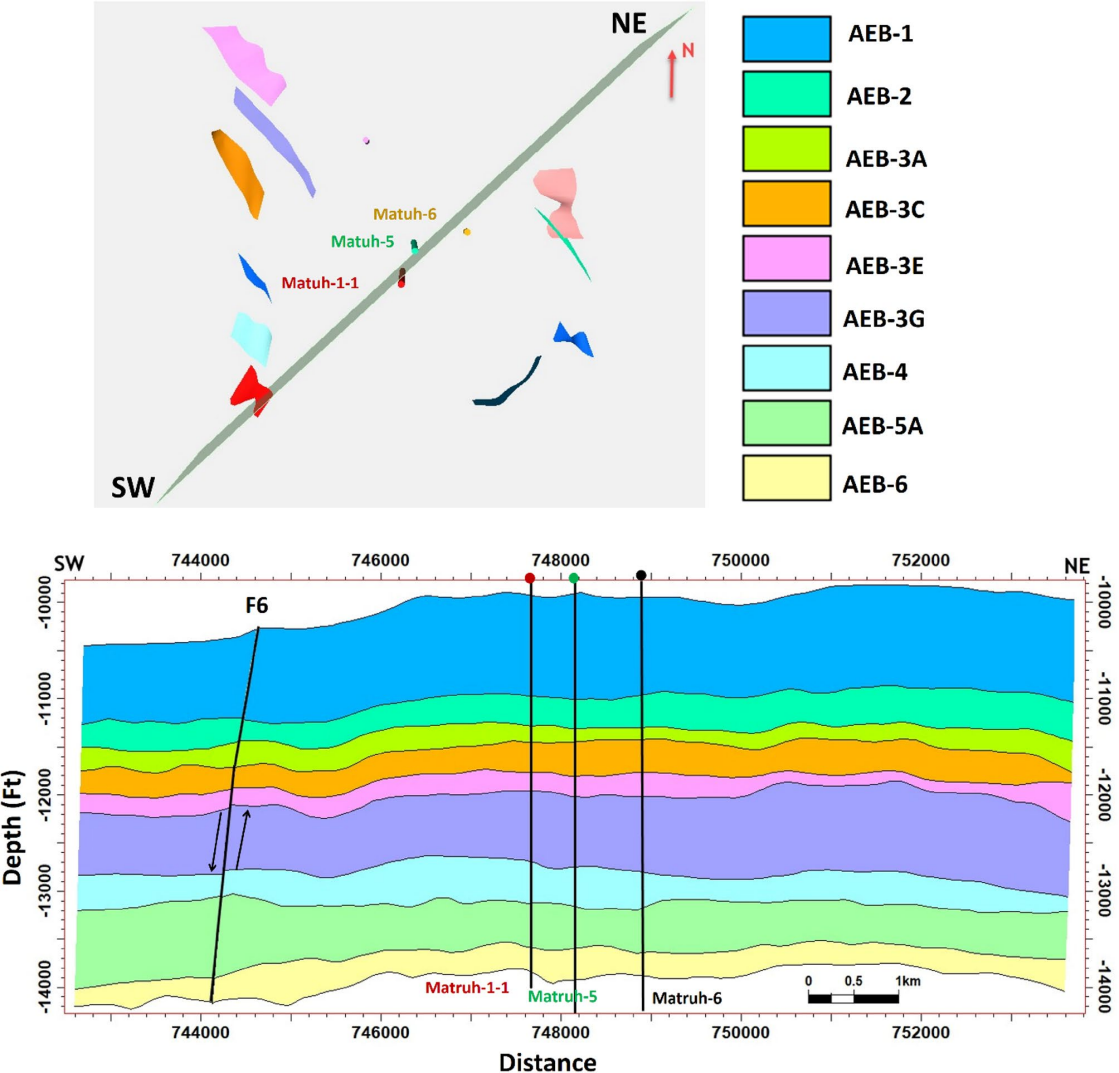
A cross section passing through the Matruh1-1, Matruh-5, and Matruh-6 wells shows the thickness distribution of the seven picked horizons of the Alam El-Bueib Formation.

The interpreted seismic sections (Figs. 14 and 15), along with the 3D structure-model intersections (Fig. 16), show that the distribution of the faults throughout the region produces both grabens and horsts. Additionally, a cross section passing through the Matruh 1-1 and Matruh-5 wells shows the thickness and the distribution of all picked horizons of the Alam El-Bueib Formation units (Fig. 17). Moreover, a cross section passing through the Matruh1-1, Matruh-5, and Matruh-6 wells shows the thickness distribution of the seven picked horizons of the Alam El-Bueib Formation (Fig. 18).

### Facies and property model

**Figure 19:**
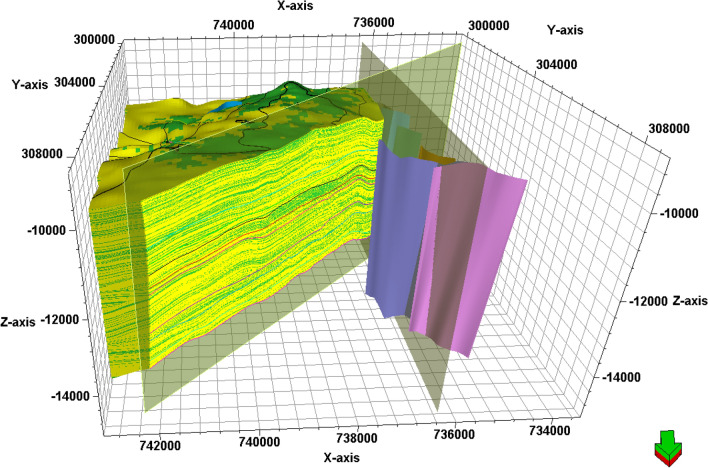
The 3D-facies model of the Alam El-Bueib Formation in the Matruh Field.

**Figure 20:**
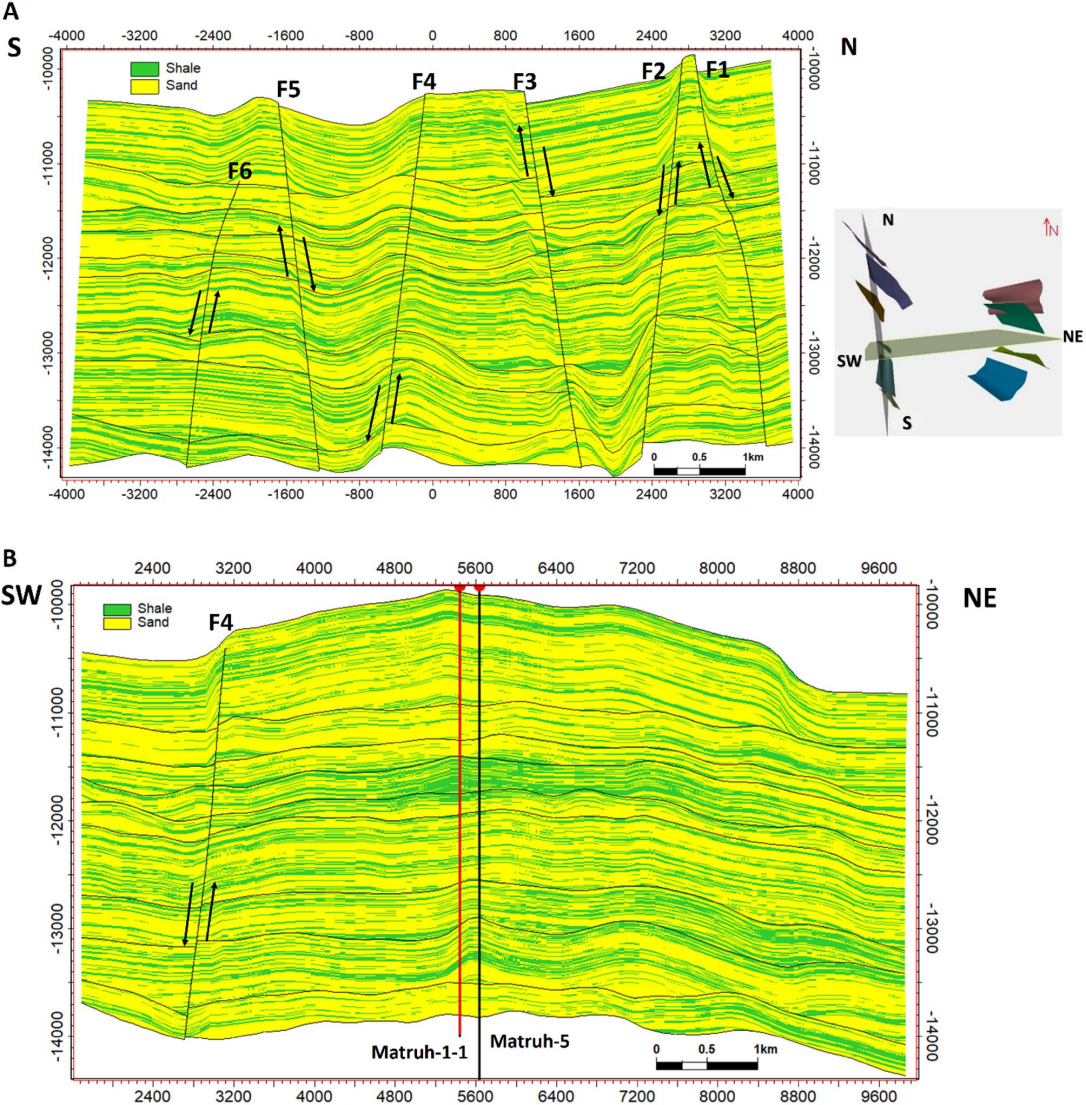
Two intersections in the direction (S–N and SW-NE) through the 3D facies model show two prominent litho-types, sandstone (yellow) and shales (green).

**Figure 21:**
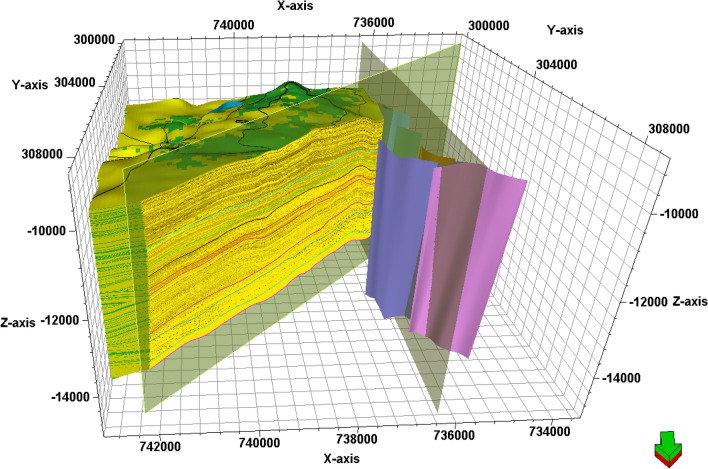
3D effective porosity model of the AEB Formation in the Matruh Field.

**Figure 22:**
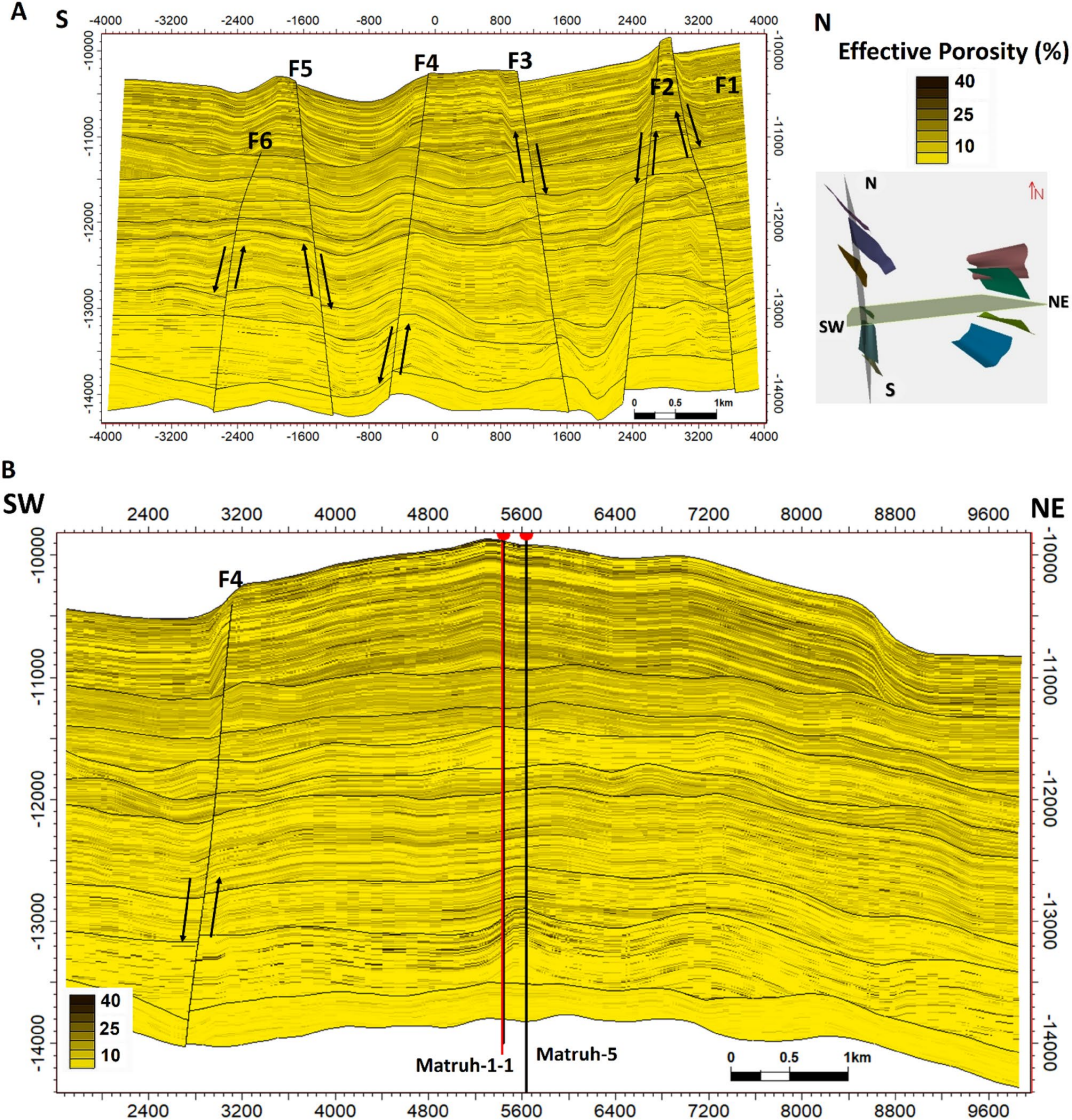
Two intersections (S–N and SW-NE) through the 3D effective porosity model of the AEB Formation showing the distribution of the effective porosity from low porosity (yellow) to high porosity (brown).

**Figure 23:**
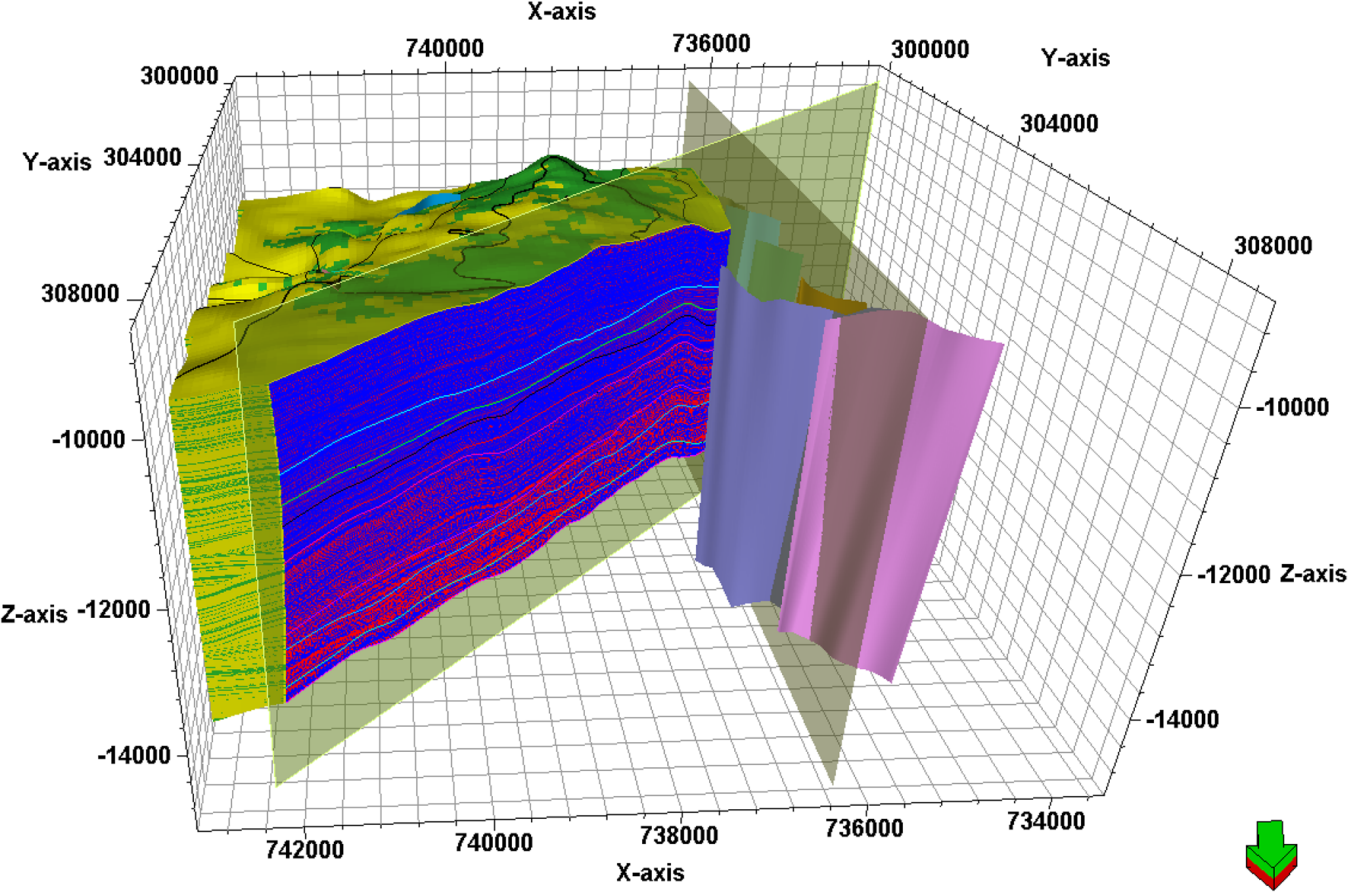
The 3D saturation model of the AEB Formation shows the distribution of water saturation from a high percentage (blue) to a low percentage (red), which corresponds to high hydrocarbon saturation (red) and low hydrocarbon saturation (blue).

**Figure 24:**
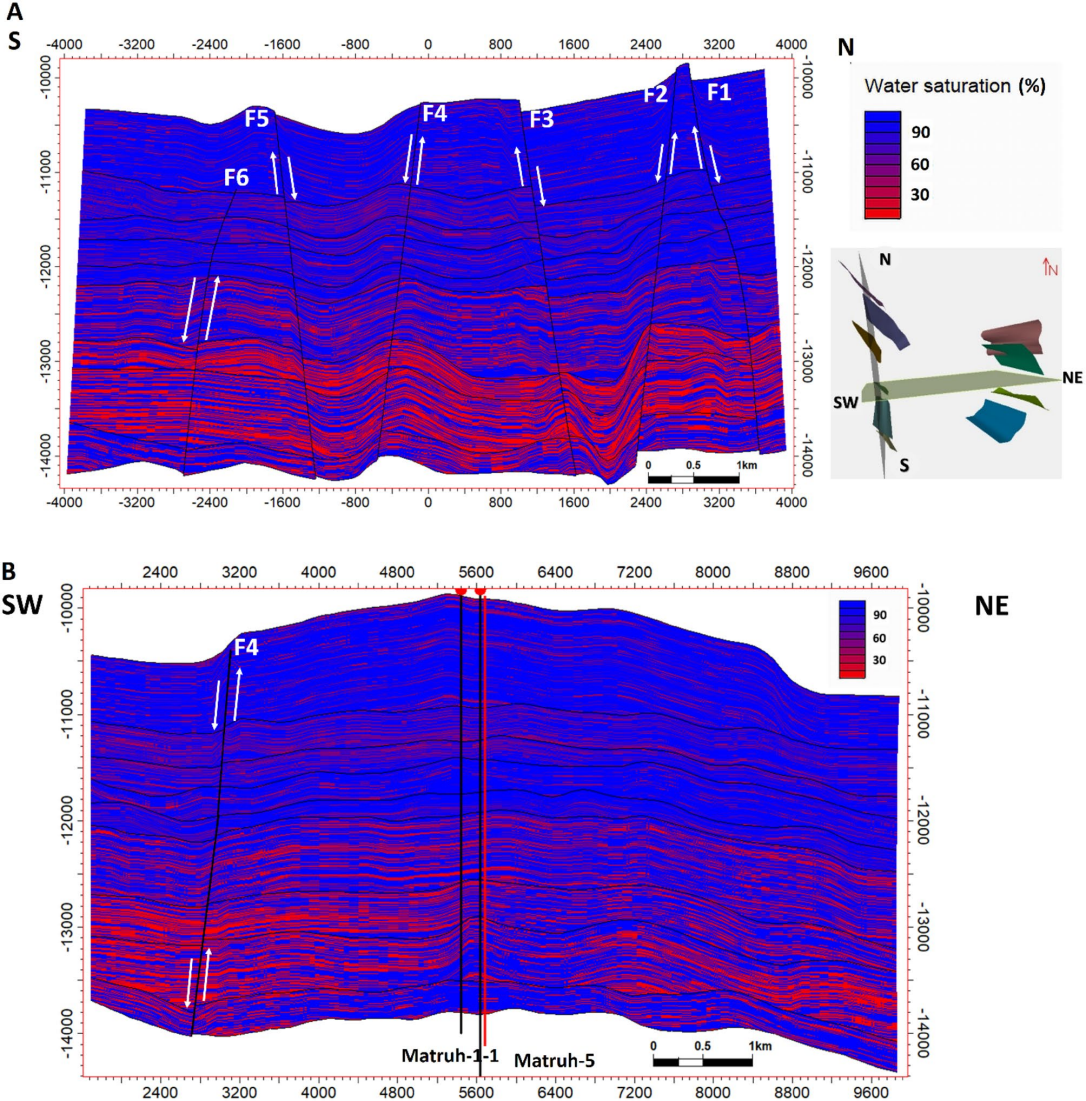
Two intersections in the directions (S–N and SW-NE) through the 3D saturation model of the AEB Formation show the distribution of the oil saturation from low (blue) to high (red).

An integrated model was constructed in Petrel to combine the four selected AEB sandstone reservoir units, cross-sections from facies, and a property model to predict the extension of hydrocarbon in the reservoir, as illustrated in Figs. 19, 20, 21, 22, 23, 24. This integrated model proves the validity of applying the 3D model in the Matruh Field.

### Model validation

**Figure 25:**
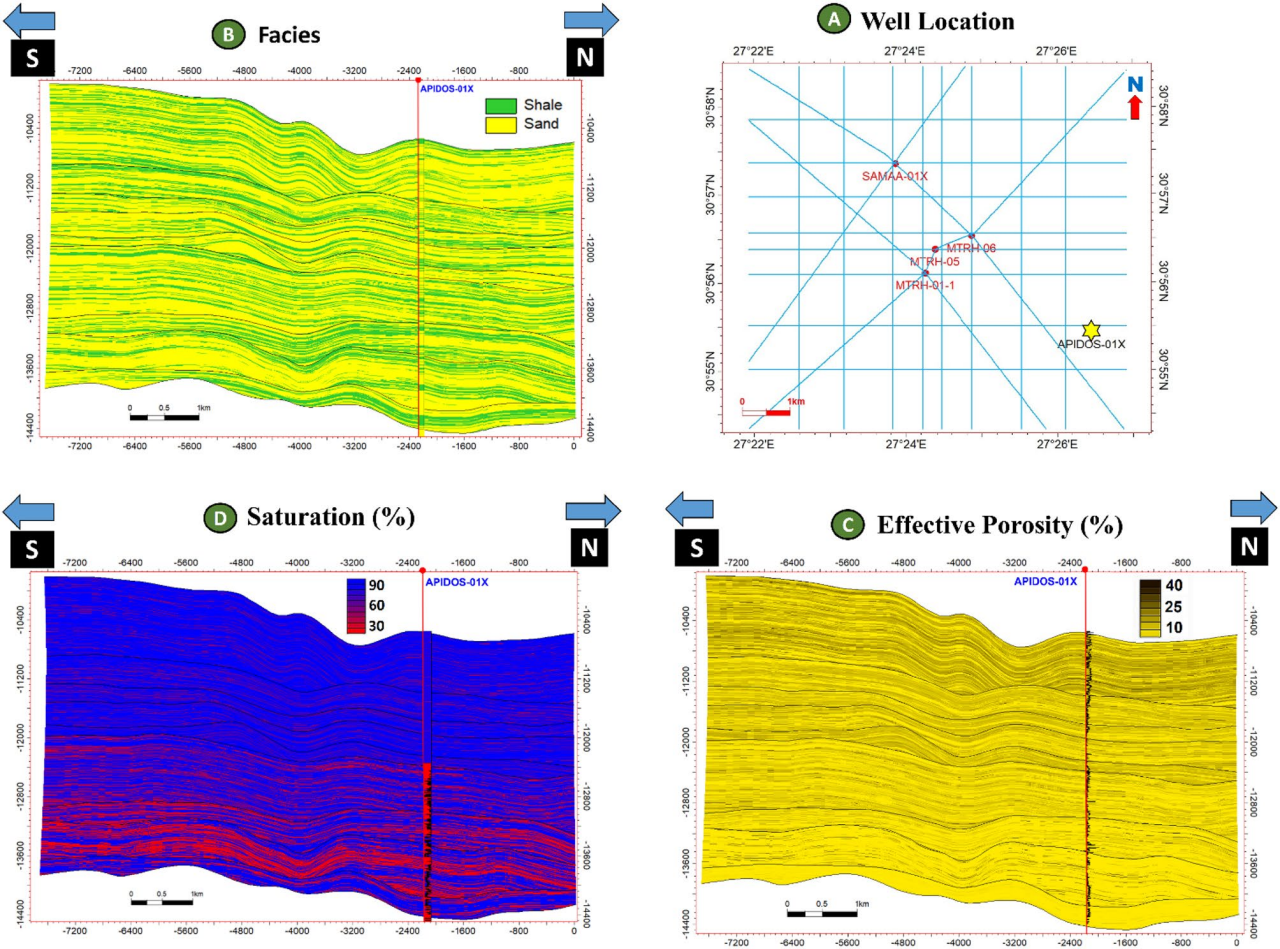
Composite figure for model validation Well, Apidose-1X shows cross sections in the direction SN in the constructed 3D facies, properties, and saturation models at the location of Apidose-1X, which illustrates a good match between the log-derived data and the built models.

The Apidose-1X well was utilized as an uncalibrated well to verify the validity of the facies, porosity, and saturation models that were produced. The facies log was obtained by using the gamma-ray log, while the estimation of effective porosity was conducted by employing neutron and density logs. Additionally, an estimation of fluid saturation was also performed. Figure 25 illustrates the intersection resulting from the convergence of the blind well and the diverse models. The log-derived facies, porosity, and saturation in the validation well exhibit a strong resemblance to those of the developed models as seen in Fig. 25.

## Discussion

The seismic interpretation of the Alam El-Bueib Formation (AEB Fm) in the Matruh Field requires identifying all the seismic facies within the formation as well as analyzing the existing structures. The AEB reservoir units in the Matruh Basin are mainly cut by normal faults, as shown in the interpreted seismic sections (Figs. 14 and 15). The provided 2D seismic lines have two principal trends, N**-**S and E-W. Seven levels of the AEB Fm were identified and picked in both directions. Two-way time structure maps were constructed for the seven levels and then converted to depth structure contour maps (Figs. 12 and 13). Moreover, the constructed structure depth maps for the members of the AEB Fm reveal that in addition to the anticline fold extending in the NE-SW direction that resulted from the Syrian Arch System event, there are a number of normal faults with two predominant NE-SW and NW–SE trends. These normal faults are structural traps, which are essential elements in the petroleum system of the field. Understanding the structures of the area is essential for optimizing the drilling and completion of wells in order to maximize hydrocarbon recovery, as faults within the formation play a major role in regulating hydrocarbon distribution and migration. The converted structure depth maps and the structure-model intersections show that in the current study, both the anticline fold and faults play a crucial role in creating AEB Fm structural traps in the Matruh Basin. An analogous study on the Alamein Field by^62^ came to the conclusion that structures in the studied area had a significant impact on the development of the reservoir rock’s thickness and trap formation. The structure identification of the Alamein Field shows that the basin inversion associated with the rifting of the Alamein area caused the abrupt increase in thickness of the AEB Fm.

In the current study, the effective porosity and facies models are quite comparable. Consequently, the property model was made to clarify the key aspects that control both models and to explain why each member of AEB Fm was modeled individually. The comprehensive examination of the AEB hydrocarbon reservoir using well logs and seismic data indicates that the neutron-density cross plots show the effect of shale content on point distribution, where the points show clusters of the same shale values. Higher shale content values pull the points down from sandstone towards limestone and dolomite lines, as shown in Fig. 9. In our study, both techniques (Vsh from gamma ray logs and neutron-density logs) were used to estimate the Vsh%. The least Vsh of both techniques was determined to be the most accurate Vsh data. The lithological heterogeneity of the AEB members affected the lateral and vertical distribution of the effective porosity and fluid saturation. Thus, four out of seven AEB Fm members (AEB-1, AEB-3A, AEB-3C, and AEB-6) show good petrophysical properties. The sandstone component of the AEB-6 member is considered to be the most favorable member in this context. However, its shaly facies is deemed to be a poor to fair oil source rock in the Matruh 1-1 well. This observation suggests that the AEB-6 member exhibits both dual source and reservoir properties. This conclusion is supported by work done on the AEB in the Jade Oil Field in the Matruh Basin, where hydrocarbon accumulation was theoretically found in the AEB-6 in the Jade-5 well by ^22^. It is important to note that the AEB Fm shows regionally heterogeneous variations in its petrophysical characteristics across the Matruh Basin. The litho-saturation panels (Fig. 10) and the 3D facies model (Figs. 19 and 20A) show that it clearly exhibits upward changes from higher shale content at its base (i.e., AEB-6 Unit) to more permeable sandy facies at the top (i.e., AEB-1) in a N-S direction along the Matruh 1-1 and Sama-1 wells. This is expected to confine generated hydrocarbons within the same impermeable shale source rock intervals, which act as unconventional reservoirs, as shown in the oil saturation model of the Matruh 1-1 and Matruh-5 wells (Fig. 24). This type of reservoir is commonly associated with drilling complications and requires special drilling strategies to have successful hydrocarbon production.

On the other hand, the AEB Fm shows an opposite trend in its petrophysical characteristics in an E-W direction. It exhibits clear upward changes from more permeable sandy facies at its base (i.e., AEB-6 Unit) to less permeable shaly facies at its top (i.e., AEB-1) along the Matruh-5 and Matruh-6 wells (Figs. 11 and 20B). Thus, the occurrence of local shale sealing rocks along the E-W direction in addition to the structural traps is expected to confine some hydrocarbon accumulations at shallower depths, as shown in the middle part of the Matruh-5 well (Fig. 24). The hydrocarbon production from this type of reservoir is expected to be easier in comparison to its counterpart along the N-S direction. Overall, the local reservoir heterogeneity shown above necessitates conducting further investigations on the AEB reservoir characteristics in other parts of the Matruh Basin to fully understand its hydrocarbon prospect.

To test the validity of our models in the current study, the Apidos-1X well was used as a blind well in the validation process of the facies, porosity, and saturation models that were derived for the studied levels of the AEB Fm. This played a crucial role because it serves as an important tool in validating the accuracy and reliability of the derived models. The good match between the log-derived data and the built models confirms the accuracy of our constructed models. This enables us to get a deeper understanding of the characteristics of the studied reservoirs and make better decisions for hydrocarbon exploration and production.

The impact of tectonic events on reservoir development and specifically its heterogeneity was also explained by work done by^63^ on a thick lower Miocene section in the Offshore North Sinai Basin. These authors^63^ reached the conclusion that the tectonic effect plays a main role in reservoir distribution, thickness, and heterogeneity. Additionally, comparable work was done by author^64^ on the Middle Jurassic and Cretaceous rocks in Tut Oilfield, Shushan Basin, through studying the composite logs for five wells. This work^64^ considered the units AEB-1, AEB-3A, 3D, and 3E of the AEB Fm as important reservoir rocks. The basin model constructed by^64^ for the organic-rich Jurassic-Cretaceous rocks indicated that AEB Fm contains some source rock intervals, which are mature and are still within the early stage of hydrocarbon generation. While reservoir evaluation of the four AEB units indicates that they are hydrocarbon-bearing and have an effective porosity mean value of 15.3% in AEB-1, 13.8% in AEB-3A, 10.7% in AEB-3D, and 14.3% in AEB-3E. These results are somehow close to the findings of our current research on the AEB Fm, particularly for the AEB-3A Unit. This indicates that AEB Fm has similar regional petrophysical characteristics across the adjacent Matruh and Shushan basins, but with local dissimilarity in other parts of the two basins is due to their relatively different tectonostratigraphic evolutions.

Regionally, to follow the effect of shale volume (Vsh) on reservoir potential, Authors^65^ studied the Lower Cretaceous of Qishn clastics reservoirs in the Sharyoof oilfield in Yemen, which revealed high hydrocarbon saturation in the S1A reservoir but almost nonexistent in the S1B reservoir due to higher shale content, 90% water saturation, and lower effective porosity. The Upper Qishn clastics subunits S1A and S1C performed best, similar to the Alam El-Bueib Formation units AEB-3A and AEB-6 in Egypt, because they have the lowest shale content.

On the other hand, authors^66^ studied the Khatatba Formation in the Matruh-Shushan Basin by interpreting thirteen 2D seismic lines and five wells. They used a 3D structure model to trace out the main structure through faults that crossed the area. They also analyzed the petrophysical properties of the Upper and Lower Safa members to see how the interpreted faults affected the main reservoirs. They used the same materials and tools as what we have used in our current research and reached the conclusion that it is imperative to construct a 3D structure model to trace out and understand the effect of faults on the studied reservoirs. However, in our current research, we additionally used the distribution of the interpreted reservoir main facies, calculated petrophysical parameters, and saturation in the 3D geologic model. We also tested the validity of our constructed model through a blind test. Authors^67^ also used the same tools and materials (i.e., composite logs and 2D seismic lines) to make reservoir characterizations for the Bahariya Formation in the Shorouk Field in the Shushan Basin. They made a correlation between the reservoir units in the 5 wells studied and interpreted 2D seismic lines by constructing 2D structure depth maps. They reached an important conclusion, which highlights the impact of the active Late Cretaceous tectonics on the development of the Bahariya Formation reservoir. Authors^68^ studied the Khataba Formation in the Apidose-1X well, which was used as a validation well in the current study in the same field, the Matruh field. They constructed a basin model, and their results reached the conclusion that the end of the Cretaceous to Early Tertiary to Late Paleocene (60–58 Ma ago) was characterized by a major uplifting phase due to tectonic inversion. Author^69^ investigated the depositional environment and paleoclimate conditions of the Middle Jurassic Khatatba Formation in Matruh-5 and Matruh-6 wells at the Matruh field in Matruh Basin. She reached the conclusion that the Tethyan rifting that occurred during the Late Jurassic and Early Cretaceous caused subsidence events in Matruh basin, which were accordingly associated with source rock maturation. By comparing our work to the work done by authors^63–69^, we can draw the conclusion that it is of prime importance to monitor the tectonics of the basin and interpret the main faults to understand their effect on reservoir thickness, distribution, and heterogeneity.

## Conclusions

Several publications investigated the hydrocarbon potential of the Early Cretaceous Alam El-Bueib (AEB) Formation in the North Western Desert of Egypt**.** However, few studies focused on the Matruh Basin. Thus, the current work investigated the petrophysical properties of the Alam El-Bueib Formation to assess its reservoir potential. The main concluding remarks of the current work are as follows:

- The Alam El-Bueib Formation is composed mainly of sandstone with several shale intercalations showing variable thicknesses and/or volumes in the studied wells.
- Four out of seven AEB members (AEB-1, AEB-3A, AEB-3C, and AEB-6) demonstrated good petrophysical properties.
- The most promising member is AEB-6, but the shaly facies part shows poor to fair source rock potential in contrast to its well-known pure reservoir character depicted from other oil fields in the Matruh Basin, which are hydrocarbon productive.
- Analysis of 2D seismic data resulted in generating depth structure maps for AEB Fm’s members, which show that the Matruh Oil Field is cut by several normal faults with NW–SE and NE-SW directions.
- Analysis of well logs and seismic data was conducted in order to develop the 3D geological and petrophysical models. The presented models illustrate the lateral and vertical distributions of the significant characteristics that determined the hydrocarbon potential of the Matruh Oil Field.
- The effective porosity and saturation models for the study area can be utilized to suggest new prospects in order to develop the Matruh Oil Field.
- 3D petrophysical modeling of AEB Fm shows the distribution of conventional reservoirs along an E-W trend and unconventional reservoirs along a N-S trend, which exemplifies its regional reservoir heterogeneity across the Matruh Basin.
- More investigations are needed to understand the regional reservoir characteristics of the AEB Fm in different parts of the Matruh Basin.

## Data Availability

The data that support the findings of this study are available from [the Egyptian General Petroleum Corporation] but restrictions apply to the availability of these data, which were used under license for the current study, and so are not publicly available. Data are however available from the corresponding author upon reasonable request and with permission of [the Egyptian General Petroleum Corporation].
